# Determinants associated with completion of postdischarge follow-up survey among multimorbid patients: a secondary analysis of the non-randomised clinical In-HospiTOOL trial

**DOI:** 10.1136/bmjopen-2025-105210

**Published:** 2025-12-12

**Authors:** Hagena Thuraisingam, Rahel Laager, Claudia Gregoriano, Philipp Schuetz, Beat Mueller, Alexander Kutz

**Affiliations:** 1 Medical University Department, Cantonal Hospital Aarau, Aarau, Switzerland; 2 Pediatrics, Luzerner Kantonsspital Kinderspital Luzern, Lucerne, Switzerland; 3 Faculty of Medicine, University of Basel, Basel, Switzerland

**Keywords:** Patient Satisfaction, Quality Improvement, Patient Reported Outcome Measures, Health Surveys, Hospital to Home Transition

## Abstract

**Importance:**

Postdischarge surveys are critical in collecting patient-reported experience measures (PREMs) and patient-reported outcome measures (PROMs), but response rates vary and are often low.

**Objective:**

To assess determinants that are associated with survey completion by phone in a complex medical care setting.

**Design:**

Secondary analysis of a prospective controlled interrupted time series analysis.

**Setting:**

As part of the non-randomised controlled In-HospiTOOL trial, a survey was conducted to gather data on PREMs and PROMs in multimorbid patients from seven hospitals in Switzerland.

**Participants:**

31 103 medical acute care hospitalisations among seven intervention hospitals who were eligible for the survey.

**Interventions:**

Over a 6-month pre-intervention phase (August 2017 through January 2018) and a subsequent 12-month intervention phase (February 2018 through January 2019), patients were contacted by phone 30 days after hospital admission.

**Main outcomes and measures:**

The primary outcome was responsiveness to the survey. We assessed group differences between responders and non-responders, and associations of patient characteristics with survey completion were estimated using generalised estimating equations.

**Results:**

Of 31 103 eligible patients, 25 557 (82.2%) completed the survey 30 days after hospital admission. Responders were marginally older than non-responders (median (IQR) age, 73 (60–82) years vs 72 (57–82); standardised mean difference (SMD), −0.08), were more likely to be Swiss (81.9% vs 74.4%; SMD, −0.18), to have private healthcare insurance (22.9% vs 17.9%; SMD, 0.12), to be living at home before admission (85.7% vs 78.6%; SMD, 0.18) and to be less frail (67.4% vs 59.1%; SMD, 0.18). A longer length of stay (OR 0.98; 95% CI 0.97 to 0.99), discharge to a non-home institution (OR 0.50; 95% CI 0.46 to 0.54) and rehospitalisation within 30 days (OR 0.78; 95% CI 0.68 to 0.89) is associated with a decreased responsiveness.

**Conclusions:**

The study shows that achieving a high survey response rate among vulnerable acute care patients is feasible, which in turn allows for the effective identification of key determinants and enhances the collection of information on patients’ experiences and outcomes.

**Trial registration number:**

ISRCTN83274049.

**Strengths and limitations of this study**

- Generalised estimating equations addressed repeat-participant correlation, though some dependence may remain.
- Large, multicentre cohort of multimorbid inpatients enabled robust patient-reported experience measure and patient-reported outcome measure assessment.
- Swiss setting ensured standardised procedures but may limit international generalisability.
- Telephone interviews achieved high response rates but required substantial staffing.
- Caregiver input increased participation, though it may not fully reflect patient views.

## Introduction

 With a stronger focus towards value-based healthcare, it became pivotal to collect patient-reported experience measures (PREMs) and patient-reported outcome measures (PROMs) to better understand the patient’s perspective on their interventions and treatments. While PREMs include information of the patient’s perspective regarding experience and satisfaction with diagnostic and therapeutic procedures, PROMs are characterised by aspects of health, such as the severity of pain or limitations in physical functioning. The optimal quantitative method for assessing various facets of health status involves directly querying patients through a standardised questionnaire or survey.^1^

The utilisation of PREMs and PROMs is advantageous not only due to their efficiency but also their cost-effectiveness and reduced burden compared with clinical measures, making them a preferred method for comprehensive and expedient data collection in healthcare. This data provides an insight into the quality of healthcare, mirrors clinical value and can be used to accelerate improvements in healthcare procedures and patient management. It also allows a direct comparison between hospitals and their provided services in terms of quality. In some cases, patient surveys are also linked to reimbursement.^2 3^

The In-HospiTOOL trial showed that the implementation of an electronic interprofessional-led discharge planning tool was associated with a decline in length of stay (LOS) without an increase in adverse outcomes.^4 5^ For this study, we were collecting PREMs and PROMs to better acknowledge the patient’s perspective on care. Within this secondary analysis of the In-HospiTOOL trial, first, we assessed the response rate on a 30-day postdischarge phone survey. Second, we identified determinants that hinder or facilitate the assessment of PREMs and PROMs in a complex medical inpatient setting.

## Methods

### Overview

This was a pre-planned secondary analysis of the non-randomised controlled In-HospiTOOL study. This study, part of the Swiss National Science Foundation’s ‘Smarter Health Care’ programme, aimed to innovate health services research and address the challenges of caring for multimorbid patients in Switzerland.^4 5^

### Study design and participants

The In-HospiTOOL (Integrative Hospital Treatment in Older Patients to Benchmark and Improve Outcome and Length of Stay) study was an investigator-initiated, multicentre quality improvement project. It examined whether implementing an electronic, interprofessional-led discharge planning tool (In-HospiTOOL) could reduce hospital LOS without compromising patient safety. The study used a controlled interrupted time series design, comparing outcomes between hospitals that implemented In-HospiTOOL (intervention group) and those that continued with standard discharge planning (control group).

Detailed information about the rationale, design and the results of the trial has been published previously.^4 5^

For secondary analyses, a survey was developed to collect data on multimorbid PREMs and PROMs. The survey included items such as satisfaction with the hospital stay and discharge, quality of life, living situation, daily activities and hospital readmission. The survey featured open, closed and multiple-choice questions. The interview is attached as online supplemental material.

Patients were recruited from seven secondary or tertiary care hospitals in Switzerland that implemented In-HospiTOOL (intervention group). Between August 2017 and January 2019, adult patients were contacted by phone 30 days after hospital admission, and surveys were conducted by study nurses. If respondents could not be reached on at least three different times and days or refused, the survey was marked unsuccessful and a reason was provided (‘patient died’, ‘patient refuses to participate’, ‘patient not reached’ or ‘other’). Family members or caregivers could respond on behalf of frail patients. The survival status of each patient was confirmed using the medical information systems of all participating study centres. If the survival status remained unclear, confirmation was requested by each patient, their family members or, if not available, by the family physician. Patients were allowed to participate multiple times, with each hospitalisation qualifying as a separate survey participation. Additionally, anonymous inpatient claims data were used to gather further demographic and outcome information.

### Outcomes

The primary outcome was responsiveness to the survey defined by the question in the survey form stating the interview took place. Secondary outcomes included reachability by phone and willingness to provide information, defined by the follow-up question asking for a reason (‘patient died’, ‘patient refuses to participate’, ‘patient not reached’ or ‘other’) when indicated the interview did not take place.

### Statistical analyses

For patient demographic information, descriptive statistics were obtained, including age, sex, nationality, insurance type and living situation. Baseline values are depicted as mean (SD), median (IQR) or number (%). Associations between patient characteristics and survey completion, reachability or willingness to provide information were estimated with generalised estimating equations providing robust SEs. This was done as our data involved correlated observations, and as we were primarily interested in estimating population-level (marginal) effects. Specifically, we fitted population-averaged logistic regression models with a binomial family, logit link, exchangeable correlation structure and robust SEs. For the willingness to provide information outcome, we used an independent correlation structure due to estimation failure caused by a large proportion of patients with only one hospitalisation. To evaluate potential differences in determinants between patients discharged home and those discharged to non-home institutions, we conducted stratified analyses by discharge setting and applied the Wald test of heterogeneity to assess the p value for interaction. We also conducted analyses to test for associations between facility discharge, LOS, rehospitalisation and survey responsiveness using the same generalised estimating equations approach. Additionally, we performed two sensitivity analyses: first, we restricted the sample to each patient’s first hospitalisation during the study period and fitted population-averaged logistic regression models with a binomial family, logit link, with an independent correlation structure; second, we fitted mixed-effects logistic regression models including all hospitalisations with a random intercept for each patient to account for within-patient correlation, using maximum likelihood estimation. Statistical significance was determined using 95% CIs, and all p values are two-sided without adjustment. Statistical analyses were performed using Stata V.17.0 (StataCorp).

## Results

### Responsiveness to the survey

A total of 31 102 eligible patients were systematically contacted by phone 30 days after hospital admission. Of these, 2232 patients (7.2%) could not be reached, and 1971 patients (6.3%) declined to provide information but were confirmed to be alive. In addition to 1342 cases (4.3%), patients were either foreign-language, deceased or the survey could not be conducted for other reasons. Thus, 5545 patients could not provide full survey information and were defined as ‘non-responders’, resulting in 25 557 completed surveys, yielding a response rate of 82.2%. Most surveys were conducted directly with the patient (83.1%), with a smaller proportion involving a family member (7.4%) or spouse (6.3%). If not conducted with the patient itself, the main reasons were cognitive impairment (29.4%), physical weakness (21.7%) or language barriers (19.7%) (online supplemental eFigure 1).

### Patient demographics

The baseline characteristics between responders and non-responders were largely similar. Responders were slightly older (median (IQR) age, 73 (60–82) years vs 72 (57–82); standardised mean difference (SMD), −0.08). Both groups were well balanced between female and male participants. Among responders, there were more Swiss residents (81.9% vs 74.4%; SMD, −0.18), more people with a supplementary healthcare insurance (22.9% vs 17.9%; SMD, 0.12) and more living at home prior to admission (89.8% vs 83.8%; SMD, 0.18). Responders had a median LOS of 6 days (IQR 3–9), compared with 6 days (IQR 4–10) for non-responders (SMD 0.14). Cardiovascular diseases and hypertension were the most common admission diagnoses in both groups, with a larger proportion among responders. Further comorbidities such as chronic kidney disease, diabetes and coronary artery disease were prevalent across both cohorts, with responders having a lower proportion of severely frail patients (3.2% vs 5.2%; SMD, 0.18) (table 1).

**Table 1:** Baseline characteristics of responders and non-responders

	Responder	Non-Responder	P value	SMD
n	n=25 557	n=5545		
Patient characteristics				
Age, median (IQR)	73.0 (60.0–82.0)	72.0 (57.0–82.0)	<0.001	−0.08
Female, n (%)	12 221 (47.8)	2722 (49.1)	0.086	0.03
Swiss citizen, n (%)	20 943 (81.9)	4125 (74.4)	<0.001	−0.18
Insurance			<0.001	0.12
Basic, n (%)	19 717 (77.1)	4552 (82.1)		
Supplementary, n (%)	5840 (22.9)	993 (17.9)		
Admission			<0.001	0.18
From home, n (%)	22 949 (89.8)	4649 (83.8)		
From facility, n (%)	2121 (8.3)	779 (14.0)		
Other, n (%)	487 (1.9)	117 (2.1)		
Number of hospitalisations			<0.001	0.20
1, n (%)	20 252 (79.2)	3941 (71.1)		
2, n (%)	4440 (17.4)	1250 (22.5)		
≥3, n (%)	865 (3.4)	354 (6.4)		
Length of stay (days), median (IQR)	6 (3–9)	6 (4–10)	<0.001	0.14
Comorbidities				
Cardiovascular, n (%)	7598 (29.7)	1293 (23.3)	<0.001	−0.15
Respiratory, n (%)	3706 (14.5)	774 (14.0)	0.30	−0.02
Oncology, n (%)	1156 (4.5)	562 (10.1)	<0.001	0.22
Gastroenterology, n (%)	2243 (8.8)	534 (9.6)	0.043	0.03
Infectious, n (%)	1921 (7.5)	412 (7.4)	0.82	0.00
Urogenital, n (%)	1078 (4.2)	245 (4.4)	0.50	0.01
Neurology, n (%)	2139 (8.4)	386 (7.0)	<0.001	−0.05
Hypertension, n (%)	14 452 (56.5)	2833 (51.1)	<0.001	−0.11
DM, n (%)	5296 (20.7)	1181 (21.3)	0.34	0.01
Heart failure, n (%)	3655 (14.3)	800 (14.4)	0.81	0.00
Coronary heart disease, n (%)	6469 (25.3)	1185 (21.4)	<0.001	−0.09
Cerebrovascular disease, n (%)	3581 (14.0)	730 (13.2)	0.098	−0.02
Chronic kidney disease, n (%)	5763 (22.5)	1333 (24.0)	0.017	0.04
Hepatopathy, n (%)	966 (3.8)	304 (5.5)	<0.001	0.08
COPD, n (%)	2528 (9.9)	548 (9.9)	0.98	0.00
Cancer, n (%)	2524 (9.9)	915 (16.5)	<0.001	0.20
Dementia, n (%)	1416 (5.5)	440 (7.9)	<0.001	0.10
Elixhauser Comorbidity Index*, mean (SD)	2.9 (2.0)	3.3 (2.1)	<0.001	0.17
Frailty Risk Score†, n (%)			<0.001	0.18
Low	17 235 (67.4)	3278 (59.1)		
Intermediate	7513 (29.4)	1978 (35.7)		
High	809 (3.2)	289 (5.2)		

* Scores range from −7 to 12 with higher scores indicating greater comorbidity.

† Scores range from 0 to 99 with higher scores indicating greater frailty.

COPD, chronic obstructive pulmonary disease; DM, diabetes mellitus; IQR, Interquartile range; SMD, standardised mean difference.

### Patient determinants associated with survey completion, phone reachability and willingness to provide information

**Figure 1:**
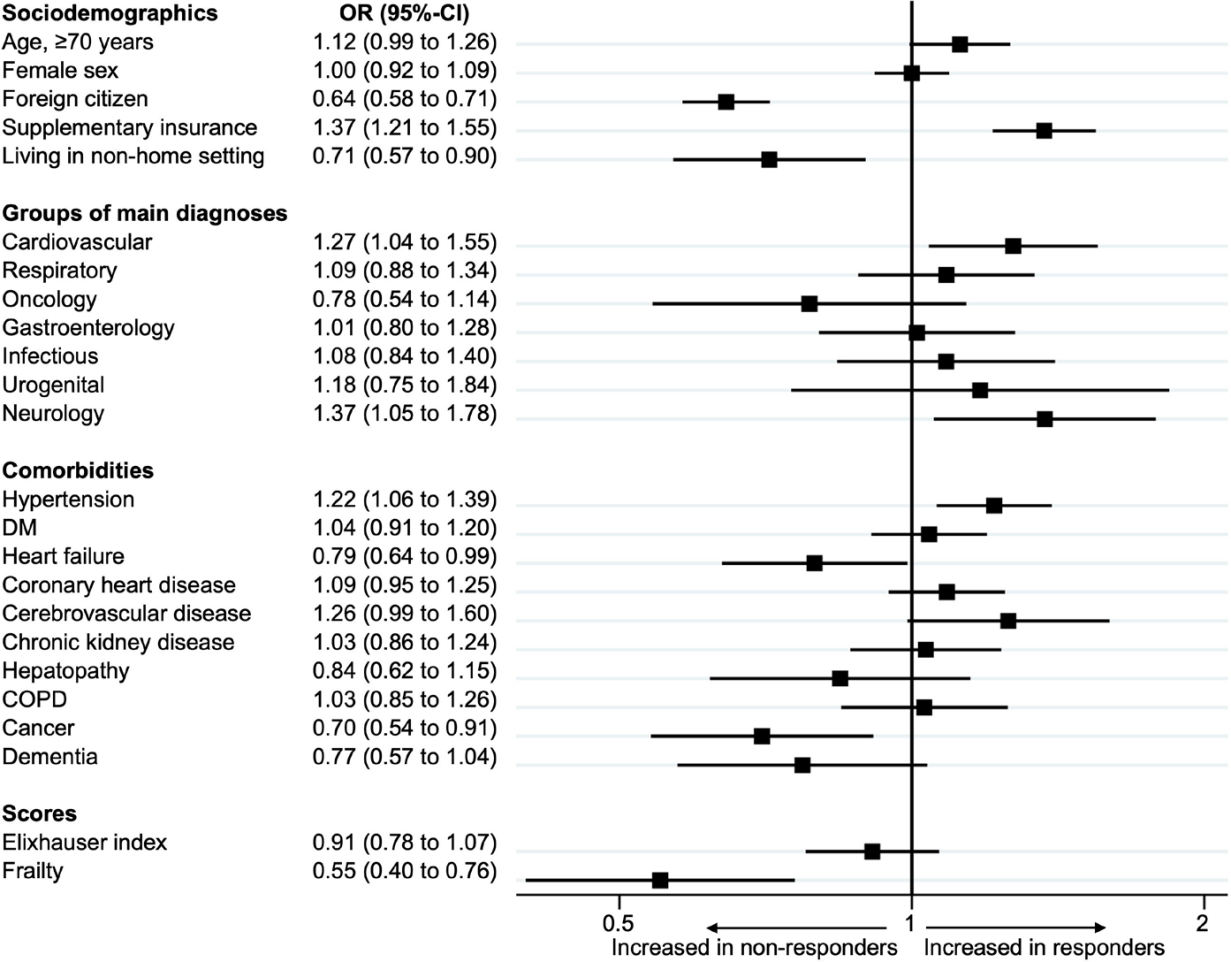
Patient determinants associated with responsiveness. The forest plot depicts graphical association between patient determinants with responders and non-responders. Determinants are on the y-axis and OR on the x-axis. COPD, chronic obstructive pulmonary disease; DM, diabetes mellitus.

The results presented in figure 1 highlight the determinants associated with the likelihood of being a survey responder among the study population. Patients who were older (reference age ≥70), those with Swiss nationality and those with a supplementary healthcare insurance were more likely to respond. Similarly, patients with known cardiovascular or neurological diseases were more likely to be responders. Conversely, patients with higher frailty scores, those with heart failure and those admitted with oncological diagnoses were less likely to be responders. Additionally, non-responders were more common among patients living in non-home institutions prior to admission.

**Figure 2:**
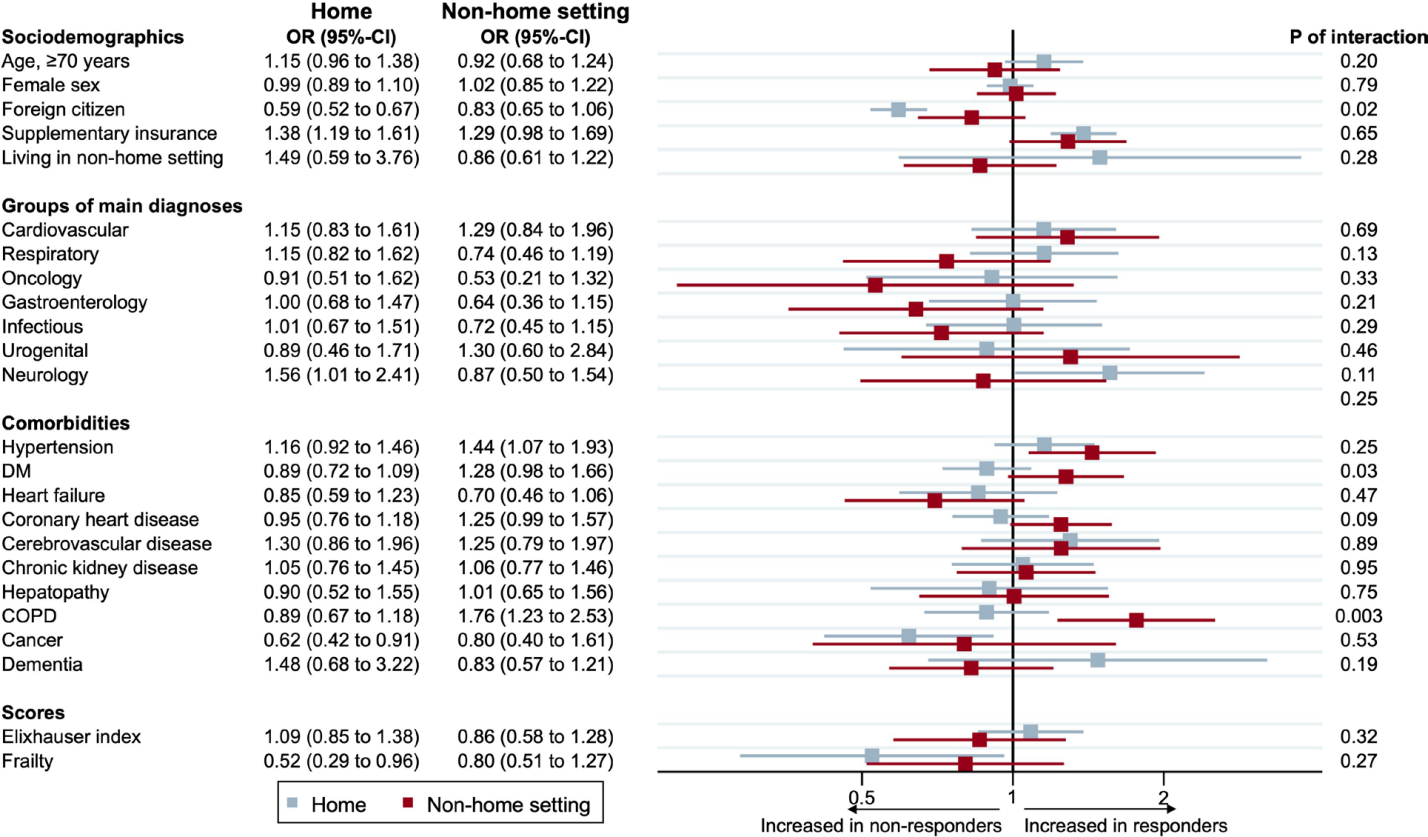
Associated factors with responsiveness between people discharged home and those discharged to a non-home institution. The forest plot illustrates the determinants associated with responsiveness, comparing discharge to home versus discharge to a non-home institution. Determinants are on the y-axis and OR on the x-axis. COPD, chronic obstructive pulmonary disease; DM, diabetes mellitus.

To assess potential differences in determinants between patients who were discharged home and in non-home institutions, we calculated the effect modification between different patient characteristics (figure 2). Baseline characteristics for patients discharged to home and patients to non-home settings can be found in online supplemental tables S2 and S3. Patients who were discharged home were more likely to participate in the survey if they had supplementary insurance or were Swiss residents. While not a significant determinant for individuals discharged home, the presence of hypertension or chronic obstructive pulmonary disease was associated with a higher likelihood of being a responder in non-home institutions.

We also assessed determinants associated with phone reachability. Like responders, individuals who were reachable by phone were older, Swiss residents, more likely to have supplementary health insurance (online supplemental eFigure 2).

Patients who were reachable but actively refused to participate in the survey were more likely to have basic health insurance, a cancer diagnosis or multiple comorbidities (online supplemental eFigure 3).

### Factors associated with responsiveness

Overall, a longer LOS, facility discharge and rehospitalisation within 30 days are associated with a decrease in responsiveness. This association is weaker in patients discharged to a non-home setting (table 2).

**Table 2:** Association of length of stay, discharge to non-home institution and 30-day rehospitalisation with responsiveness

Factors associated with responsiveness	OR (95% CI)	P value
Overall		
Length of stay	0.98 (0.97 to 0.99)	<0.001
Discharge to a non-home setting	0.50 (0.46 to 0.54)	<0.001
Rehospitalisation in 30 days	0.78 (0.68 to 0.89)	<0.001
Discharged to home		
Length of stay	1.01 (0.99 to 1.03)	0.356
Rehospitalisation in 30 days	0.69 (0.57 to 0.83)	<0.001
Discharged to non-home setting		
Length of stay	1.00 (0.99 to 1.01)	0.70
Rehospitalisation in 30 days	0.80 (0.58 to 1.11)	0.18

Model: crude population-averaged logistic regression (xtgee), binomial family, logit link, exchangable correlation structure, robust SEs.

### Sensitivity analyses

When only including each patient’s first hospitalisation over the study period 24 193 patients were contacted, of which 20 252 (83.7%) were responders. Among the 3941 non-responders, 1666 (42.2%) could not be reached, 1339 (34.0%) refused to participate, 218 (5.5%) did not speak German, 549 (13.9%) were diseased and 169 (4.2%) were not able to complete the interview for other reasons. Patient demographics did not differ relevantly when only including the first hospitalisation compared with all hospitalisations (online supplemental table S1).

Characteristics for responsiveness, reachability and refusal to provide information remained robust. However, patients with diabetes are more likely to be a responder during the first hospitalisation only. Furthermore, patients hospitalised for cardiological or neurological reasons were more likely to be reached by telephone after their first hospitalisation (online supplemental eFigures 4–6).

Results from the second sensitivity analysis using mixed-effects logistic regression, accounting for within-patient correlation, were consistent with the main findings, although CIs were wider, further supporting the robustness of the results (online supplemental eFigures 7–10).

## Discussion

In this study, we analysed patient characteristics and survey completion rates among 31 103 eligible participants. In general, the response rate was high with 82.2%. Responders were generally older, Swiss residents and had lower multimorbidity and frailty scores. They were more likely to have cardiovascular or neurological diseases, while non-responders were more commonly frail, had dementia or had oncological diagnoses. Responders had shorter hospital stays, were less likely to be discharged to a non-home institution and had a lower relative risk for 30-day rehospitalisation compared with non-responders, particularly among those discharged home.

In previous studies, survey response rates have varied widely, often ranging from 5% to 70%, depending on the study design, the mode of contact (eg, mailing, phone, visits) and population targeted.^6^,^9^ This variability can lead to significant biases, particularly when only the most satisfied or dissatisfied patients choose to respond, leaving a large portion of the population under-represented. Such response bias limits the ability to draw comprehensive conclusions about the entire study population, as the feedback may not accurately reflect the representative experiences of the majority or the whole study population.

For example, studies have shown that lower response rates can undermine the validity of survey findings, as the data may not represent the full spectrum of patient experiences, especially in critical areas such as patient satisfaction and outcomes.^10^ To counteract this, it is crucial to achieve as high a response rate as possible, ensuring a more accurate and holistic view of the patient population. This is particularly important in studies like the In-HospiTOOL study,^5^ where understanding both ‘hard’ outcomes and PREMs and PROMs is essential for evaluating the effectiveness of interventions.

Despite the challenges, our study achieved a notably high response rate of 82.2% using telephone interviews, which compares favourably with the response rates reported in the literature. While telephone interviews require more resources, they have proven effective in obtaining reliable data, as seen in our study. Although alternative methods like mail or email surveys may reduce costs, they typically do not achieve the same level of response, highlighting the trade-off between resource investment and data reliability.^8 11^

The completion of patient surveys is influenced by various demographic and clinical factors, which can significantly affect the quality and reliability of the data collected. In our study, factors positively associated with survey completion included older age, Swiss residency, having supplementary health insurance, discharge to non-post-acute care institutions and a lower frailty score. These findings are consistent with previous research that has identified similar trends. For example, patients with depression have also been found to be more likely to participate in surveys, possibly due to their frequent interactions with healthcare services and a heightened interest in their care outcomes.^12^

Conversely, factors associated with survey non-completion in our study included being a foreigner, living in a non-home institution prior to hospital admission, having a high frailty score and having an oncological diagnosis. These findings align with existing literature, where non-white ethnicity, younger age, male sex, unmarried status, low socioeconomic status, low income, ethnic minority status, lower educational levels, pain and opioid use have all been associated with lower survey participation rates.^613^,^15^ The reluctance of patients with high frailty scores or oncological diagnoses to participate may be due to their impaired medical condition, which diminishes their interest or capacity to engage in surveys.

Understanding these factors is crucial for improving survey response rates, particularly as quality measures in healthcare become increasingly important. Surveys are often used to assess patient-reported experiences and outcomes, which are vital for evaluating the effectiveness of healthcare interventions and for continuous quality improvement. However, the presence of response biases—where only certain subgroups are over-represented—can skew results and reduce the generalisability of findings.^16^

Although our study found that responders generally had shorter lengths of stay, it is important to note that in other studies, such as those involving patients undergoing hip arthroplasty, survey participation was not related to the length of hospital stay.^17^ This suggests that while certain determinants are consistently associated with survey completion, others may vary depending on the specific patient population or clinical context.

Subgroup analyses further highlighted that among patients discharged home, factors such as Swiss residency, older age and a lower frailty score were positively associated with survey responsiveness. These insights are valuable for tailoring survey strategies to improve response rates, particularly in populations that are typically under-represented.

This study has limitations. First, the study was conducted exclusively in Switzerland, limiting the generalisability of the findings to countries with larger or different populations. The focus on multimorbid patients further restricts the applicability of these results to other patient groups. However, this focus is particularly important as more information on PREMs and PROMs is critically needed in this vulnerable population, aligning closely with our primary objective in conducting the In-HospiTOOL study. Second, the use of telephone interviews, conducted by research assistants, required significant staffing resources due to the large number of participants. While an email, smartphone or postal survey would have been less expensive, it is uncertain and in our opinion doubtful whether such methods would have achieved a similarly high response rate. Additionally, the inability to blind study nurses may have introduced reporting and participation biases.^5^ Language barriers also posed challenges, as non-German-speaking participants might have encountered misunderstandings during the telephone interviews. Third, PROMs are inherently limited for individuals unable to articulate their health experiences due to severe illness or cognitive impairment. While input from caregivers or relatives can be considered, it does not fully substitute for self-reported data. Moreover, patients’ recall of their hospital experiences may be subject to memory biases.^1^ Additionally, surveys were conducted 30 days after hospital admission rather than after discharge, which may affect the comparability of responses due to varying follow-up times depending on patients’ LOS; however, this applied equally to both groups. Lastly, this study was not designed to include multiple survey methods, which would have allowed for a comparison of the factors influencing response rates and data quality.

## Conclusions

In conclusion, several factors were associated with the completion of PREMs and PROMs surveys in multimorbid patients, including older age, Swiss residency, supplementary health insurance, discharge to a non-post-acute care institution and lower frailty level. Additionally, responders generally had shorter hospital stays compared with non-responders. Understanding these patient-related factors may help hospitals and clinicians tailor future surveys to improve response rates and, consequently, enhance patient care and outcomes. By considering these factors, healthcare providers can better engage patients in providing valuable feedback, leading to more accurate assessments of patient experiences and outcomes.

## Supplementary material

10.1136/bmjopen-2025-105210 online supplemental file 1

10.1136/bmjopen-2025-105210 online supplemental file 2

## Data Availability

Data are available upon reasonable request.
