# Supplementary material for: Determinants associated with completion of postdischarge follow-up survey among multimorbid patients: a secondary analysis of the non-randomised clinical In-HospiTOOL trial
Source: BMJ Open. 2025 Dec 12;15(12):e105210. doi: 10.1136/bmjopen-2025-105210 (PMC12706230; doi:10.1136/bmjopen-2025-105210)
Supplement: online supplemental file 1 [file bmjopen-15-12-s001.docx]

**Supplementary Appendix**

This appendix has been provided by the authors to give readers additional information about their work.

Supplement to: Thuraisingam H. et al., **Determinants associated with completion of postdischarge follow-up survey among multimorbid patients – a secondary analysis of the nonrandomized clinical In-HospiTOOL trial**

**Table of contents**

[eFigure 1 – Reasons for a response by another individual 3](#_Toc213164539)

[eFigure 2 – Patient determinants associated with reachability by phone 4](#_Toc213164540)

[eFigure 3 – Patient determinants associated with willingness to provide information 5](#_Toc213164541)

[eFigure 4 – Patient determinants associated with responsiveness after the first hospitalization only 6](#_Toc213164542)

[eFigure 5 – Patient determinants associated with reachability by phone after the first hospitalization only 7](#_Toc213164543)

[eFigure 6 – Patient determinants associated with refusal to provide information after the first hospitalization only 8](#_Toc213164544)

[eFigure 7 – Patient determinants associated with responsiveness – mixed-effects model 9](#_Toc213164545)

[eFigure 8 – Associated factors with responsiveness between people discharged home and those discharged to a non-home institution – mixed-effects model 10](#_Toc213164546)

[eFigure 9 – Patient determinants associated with reachability by phone – mixed-effects model 11](#_Toc213164547)

[eFigure 10 – Patient determinants associated with refusal to provide information – mixed effects model 12](#_Toc213164548)

[Table S1 Baseline characteristics of responders and non-responders after the first hospitalization in the study period only 13](#_Toc213164549)

[Table S2 Baseline characteristics of responders and non-responders with discharge to home 14](#_Toc213164550)

[Table S3 Baseline characteristics of responders and non-responders with discharge to non-home setting 15](#_Toc213164551)

# **eFigure 1 – Reasons for a response by another individual**


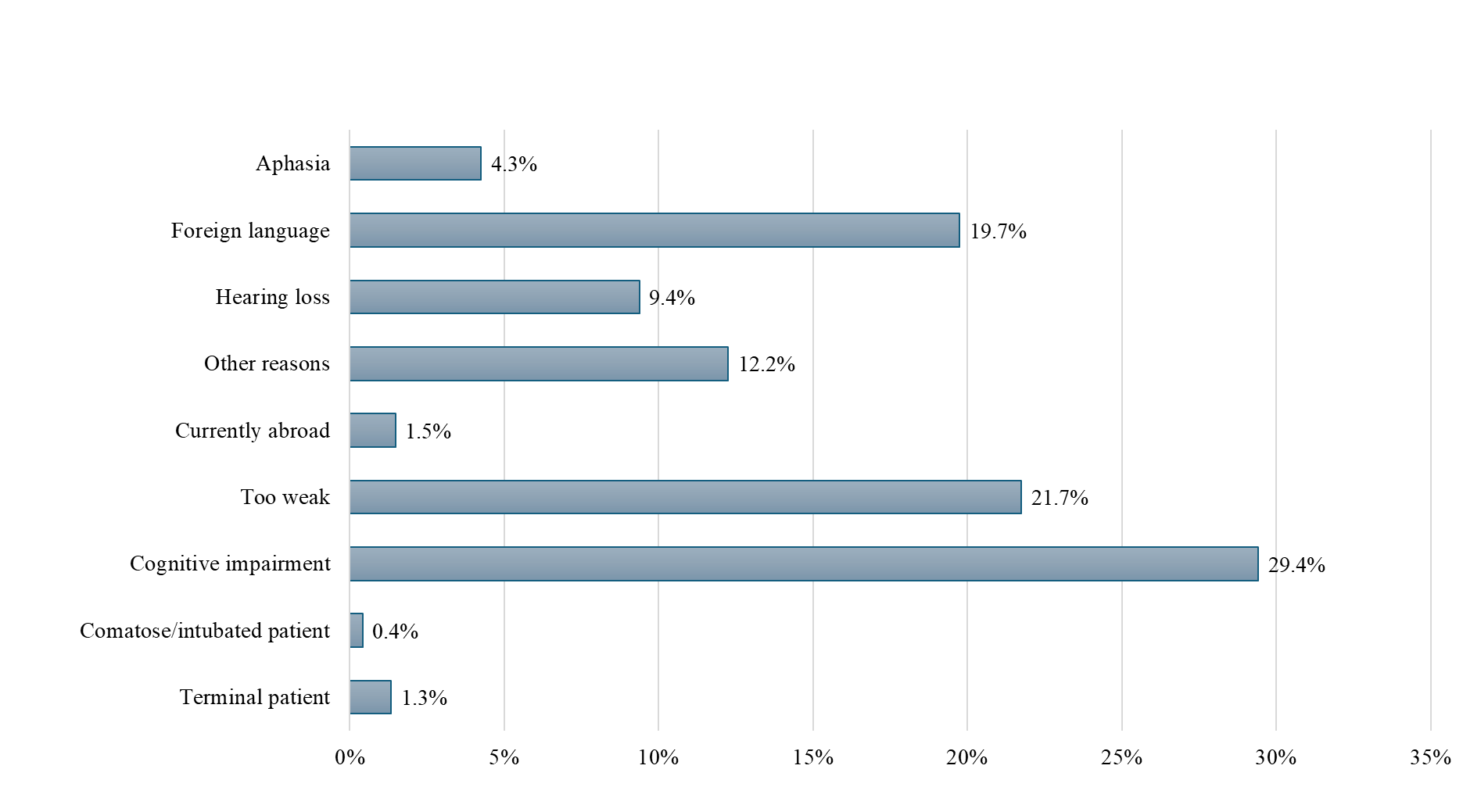


The bar chart shows the reasons for not conducting the interview with the patient. The percentages are shown in each bar.

# **eFigure 2 – Patient determinants associated with reachability by phone**


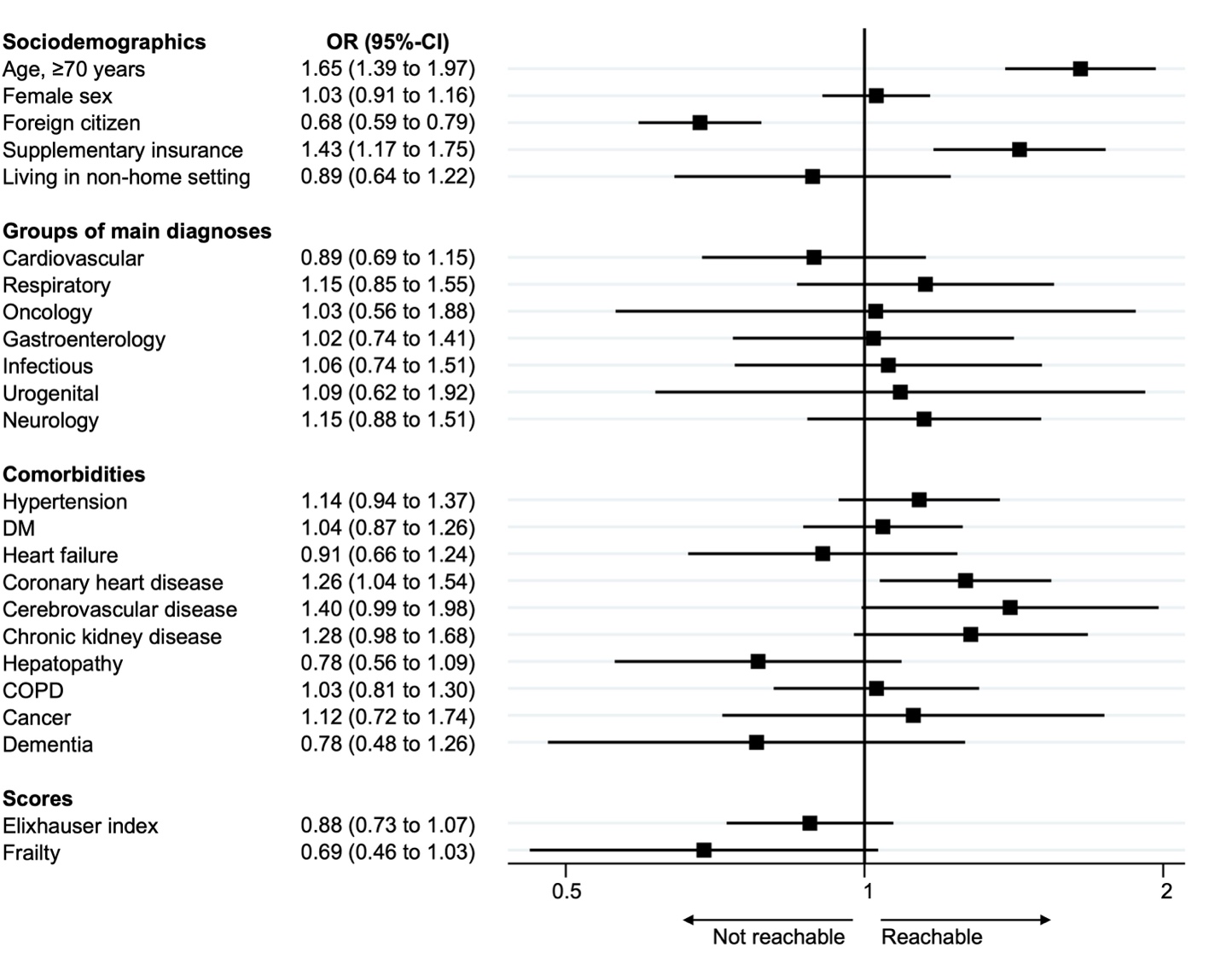


The forest plot depicts graphical association between patient determinants with people who could be reached by phone and those who were not reachable. Determinants are on the y-axis and odds ratio on the x-axis.

Model specification: Population-averaged logistic regression (xtgee, binomial family, logit link, exchangeable correlation, robust SE).

# **eFigure 3 – Patient determinants associated with willingness to provide information**


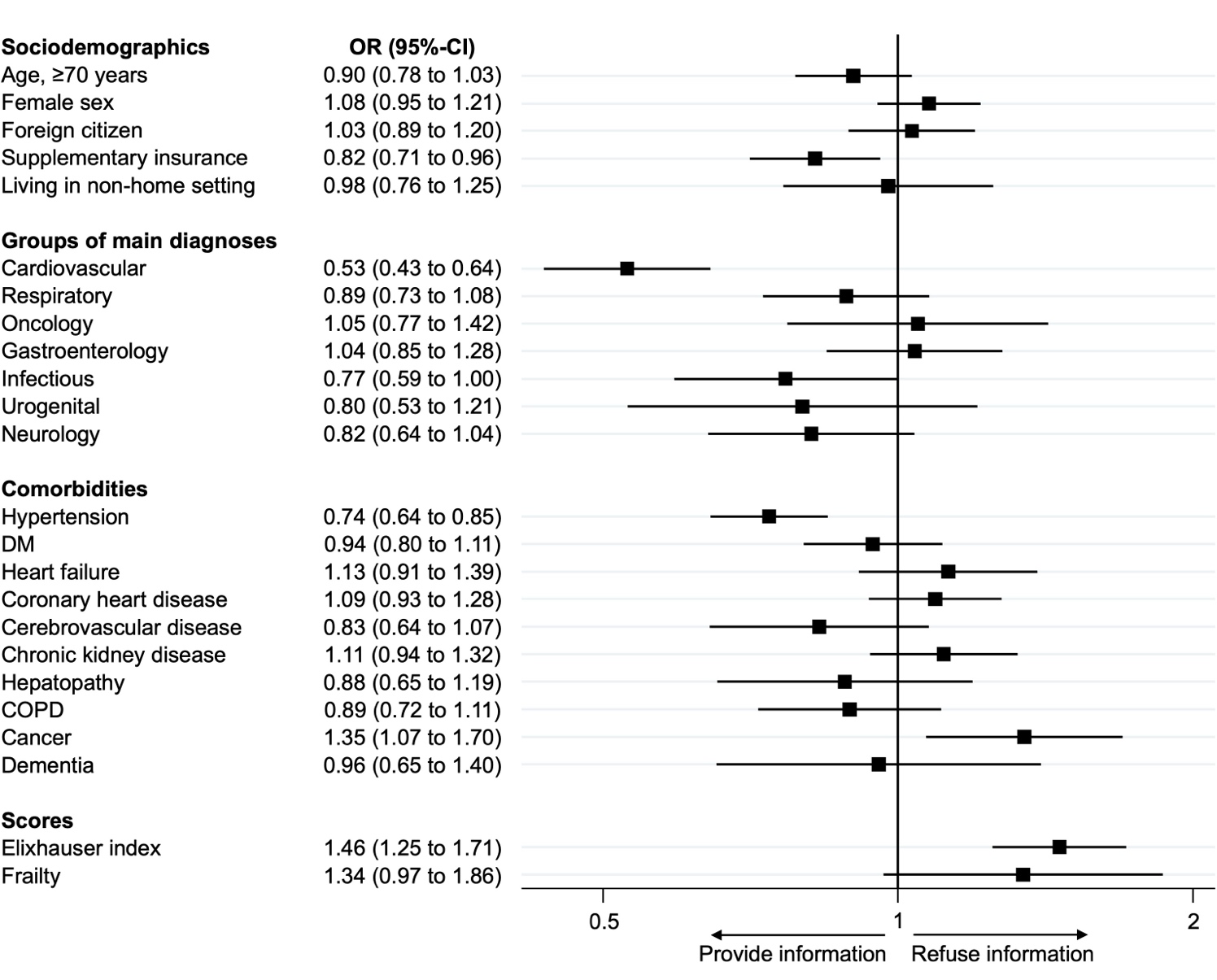


The forest plot depicts graphical association between patient determinants with people who were willing to provide information and those who refused. Determinants are on the y-axis and odds ratio on the x-axis.

Model specification: Population-averaged logistic regression (xtgee, binomial family, logit link, independent correlation, robust SE).

# **eFigure 4 – Patient determinants associated with responsiveness after the first hospitalization only**


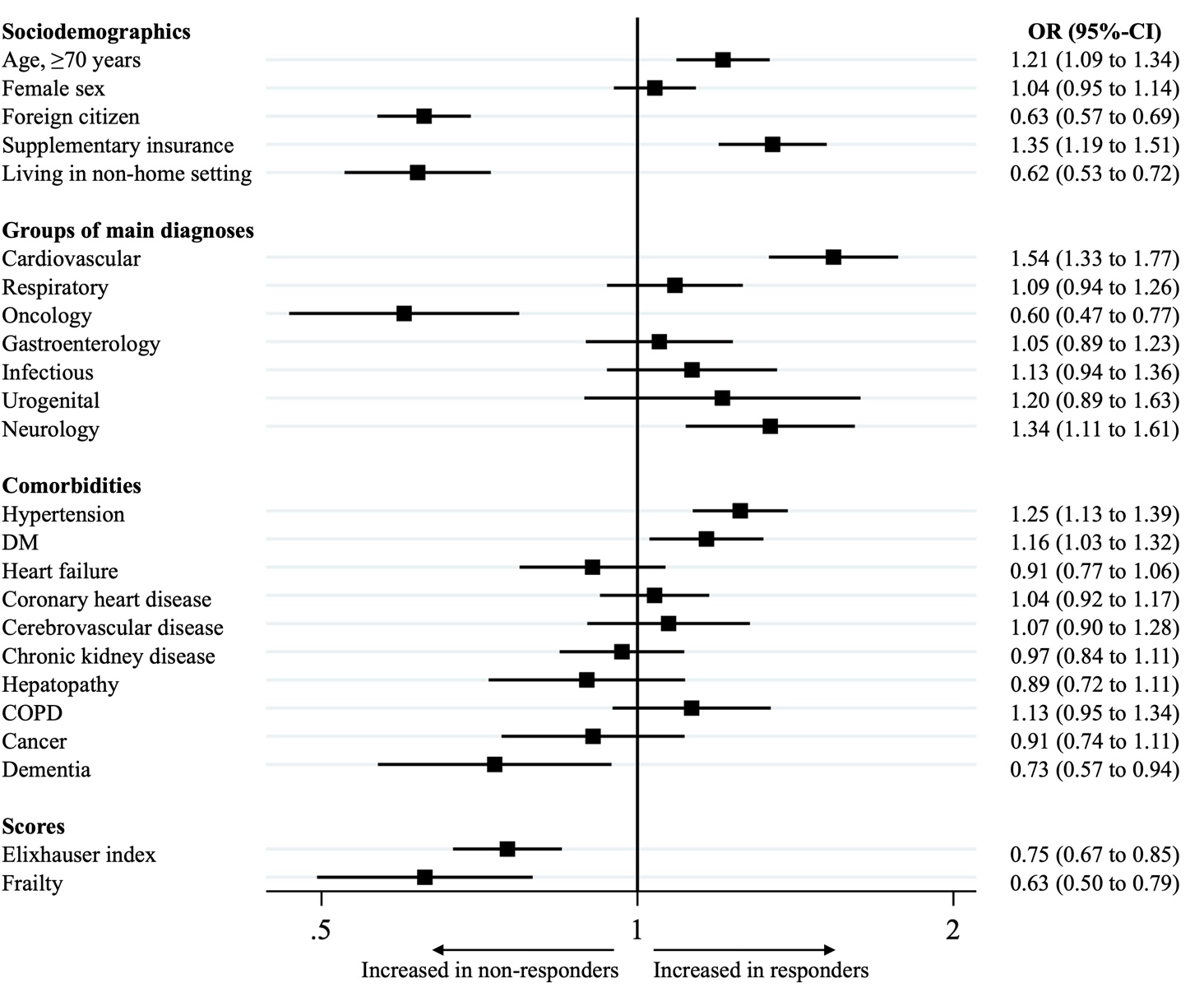


The forest plot depicts graphical association between patient determinants with responders and non-responders after the first hospitalization in the study period only. Determinants are on the y-axis and odds ratio on the x-axis.

Model specification: Population-averaged logistic regression (xtgee, binomial family, logit link, independent correlation, robust SE).

# **eFigure 5 – Patient determinants associated with reachability by phone after the first hospitalization only**

**
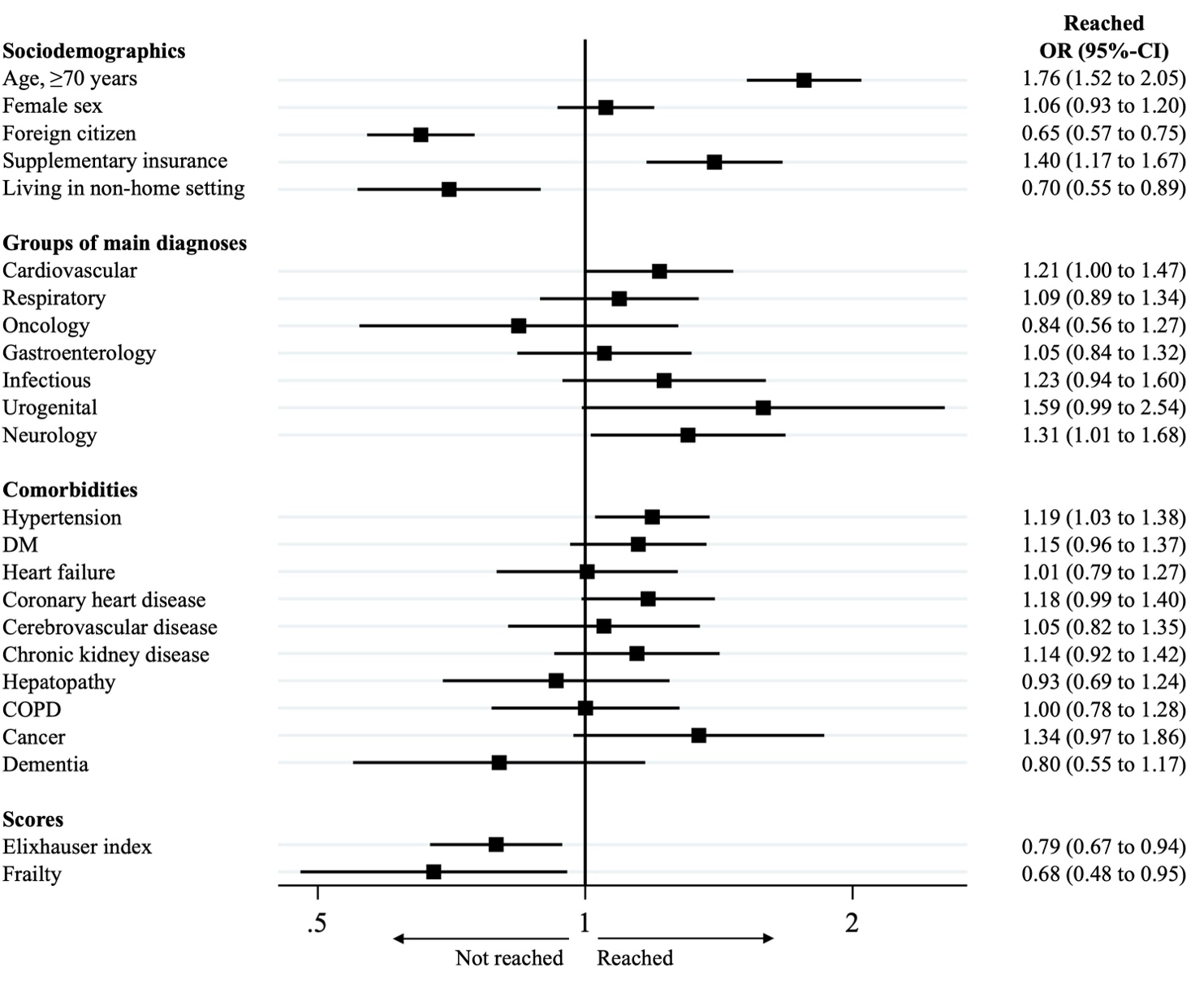
**

The forest plot depicts graphical association between patient determinants with people who could be reached by phone and those who were not reachable after the first hospitalization in the study period only. Determinants are on the y-axis and odds ratio on the x-axis.

Model specification: Population-averaged logistic regression (xtgee, binomial family, logit link, independent correlation, robust SE).

# **eFigure 6 – Patient determinants associated with refusal to provide information after the first hospitalization only**


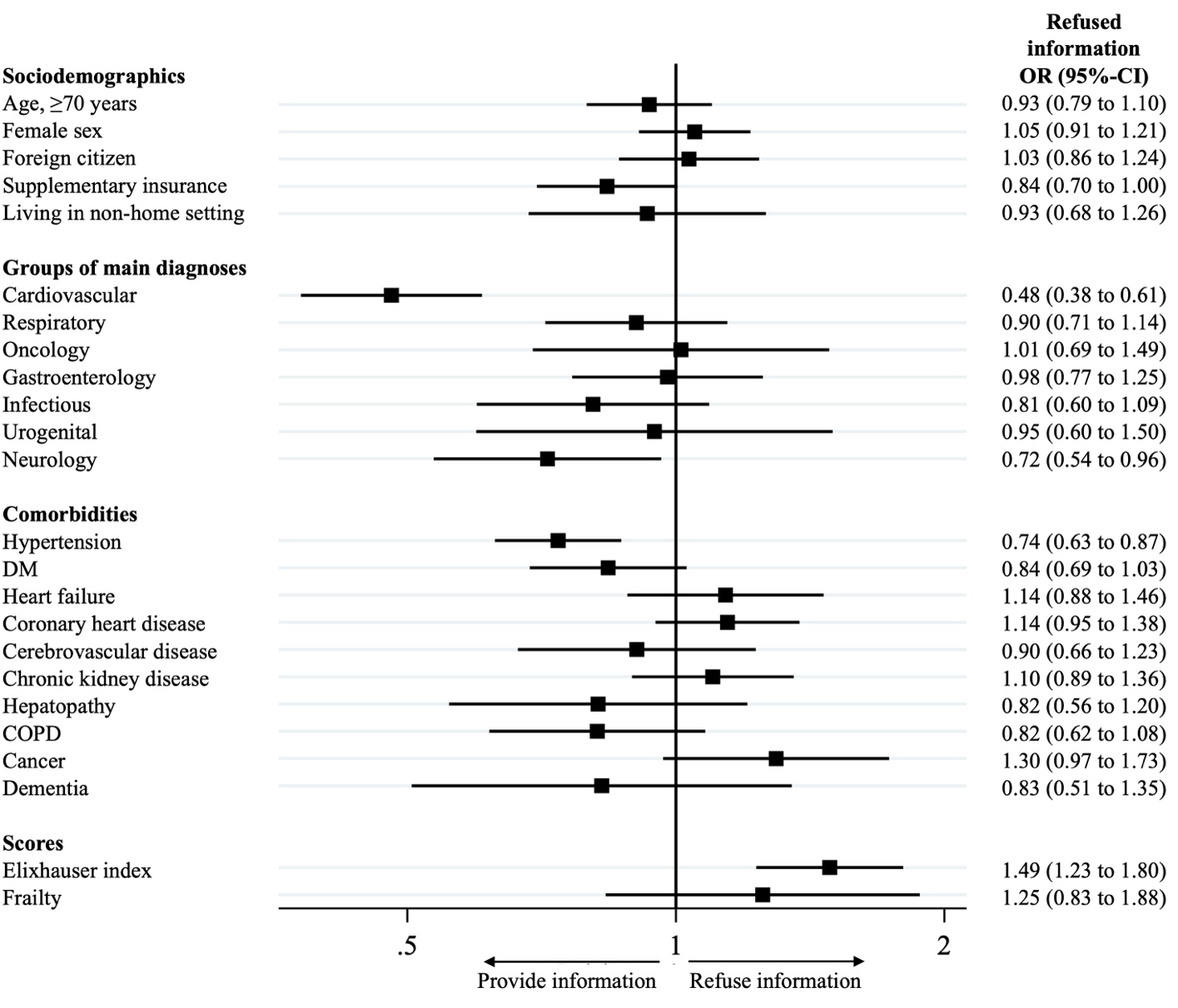


The forest plot depicts graphical association between patient determinants with people who were willing to provide information and those who refused after the first hospitalization in the study period only. Determinants are on the y-axis and odds ratio on the x-axis.

Model specification: Population-averaged logistic regression (xtgee, binomial family, logit link, independent correlation, robust SE).

# **eFigure 7 – Patient determinants associated with responsiveness – mixed-effects model**


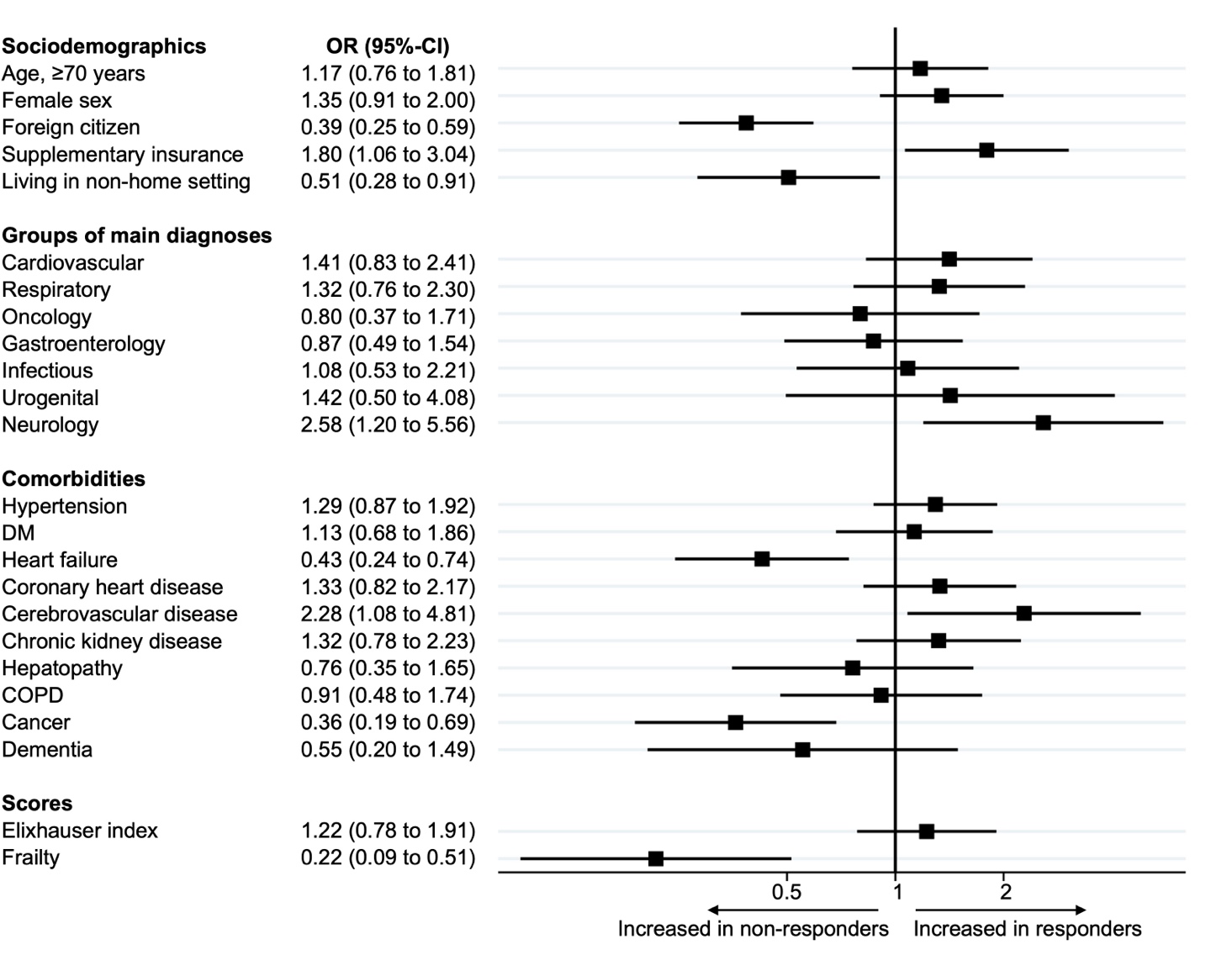


The forest plot depicts graphical association between patient determinants with responders and non-responders. Determinants are on the y-axis and odds ratio on the x-axis.

Model specification: Mixed-effects logistic regression with patient-specific random intercepts.

# **eFigure 8 – Associated factors with responsiveness between people discharged home and those discharged to a non-home institution – mixed-effects model**


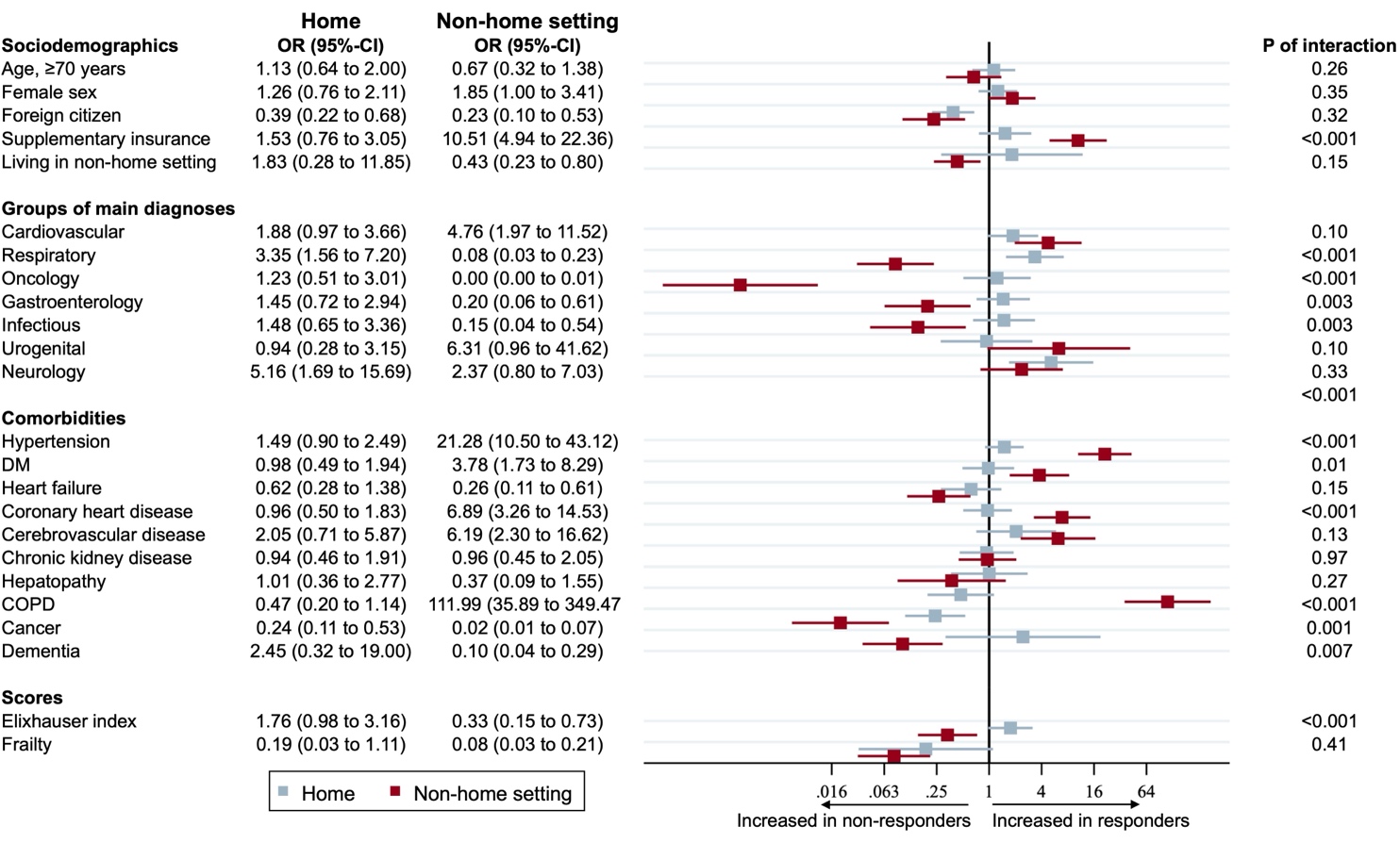


The forest plot illustrates the determinants associated with responsiveness, comparing discharge to home versus discharge to a non-home institution. Determinants are on the y-axis and odds ratio on the x-axis.

Model specification: Mixed-effects logistic regression with patient-specific random intercepts.

# **eFigure 9 – Patient determinants associated with reachability by phone – mixed-effects model**


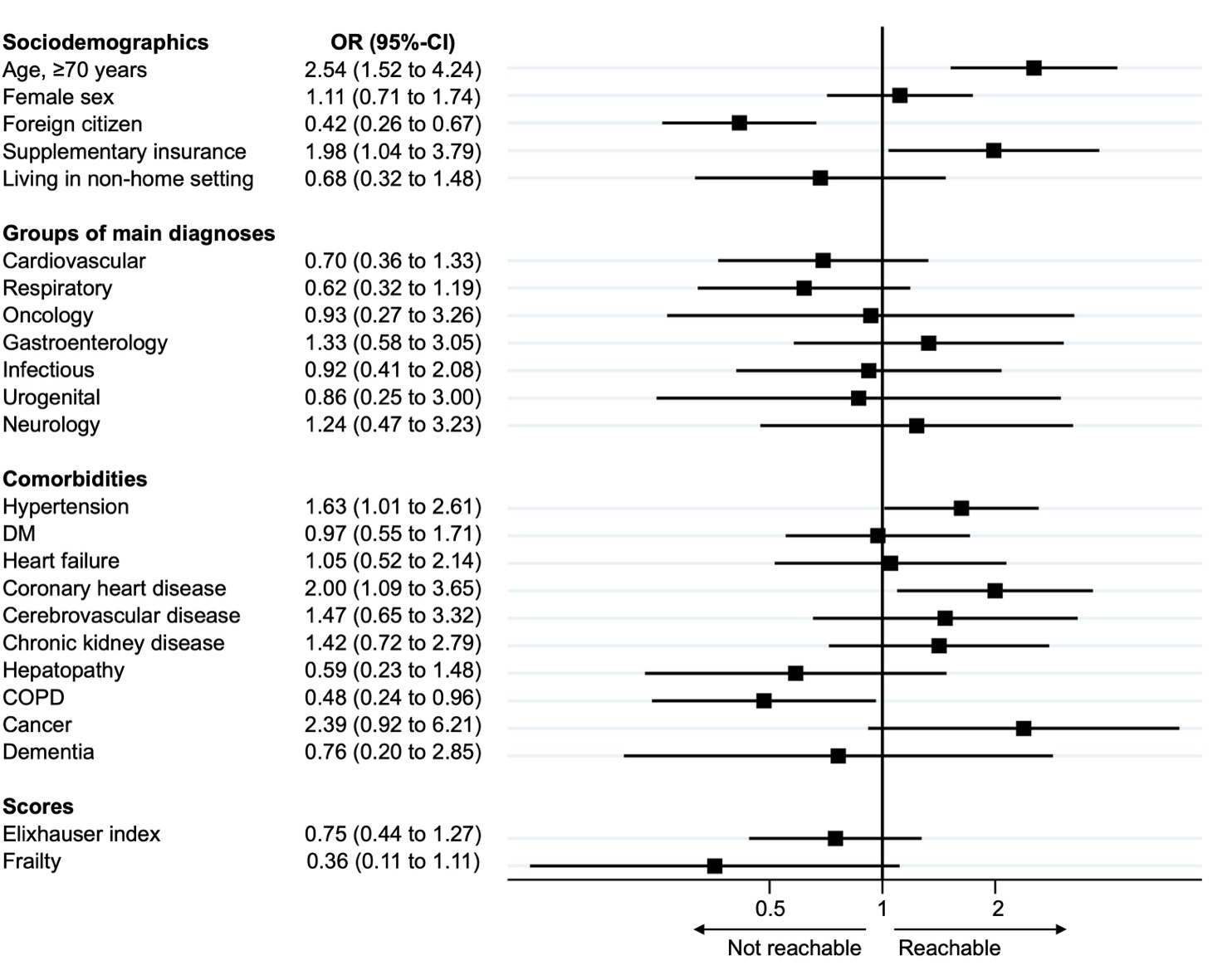


The forest plot depicts graphical association between patient determinants with people who could be reached by phone and those who were not reachable. Determinants are on the y-axis and odds ratio on the x-axis.

Model specification: Mixed-effects logistic regression with patient-specific random intercepts.

# **eFigure 10 – Patient determinants associated with refusal to provide information – mixed effects model**


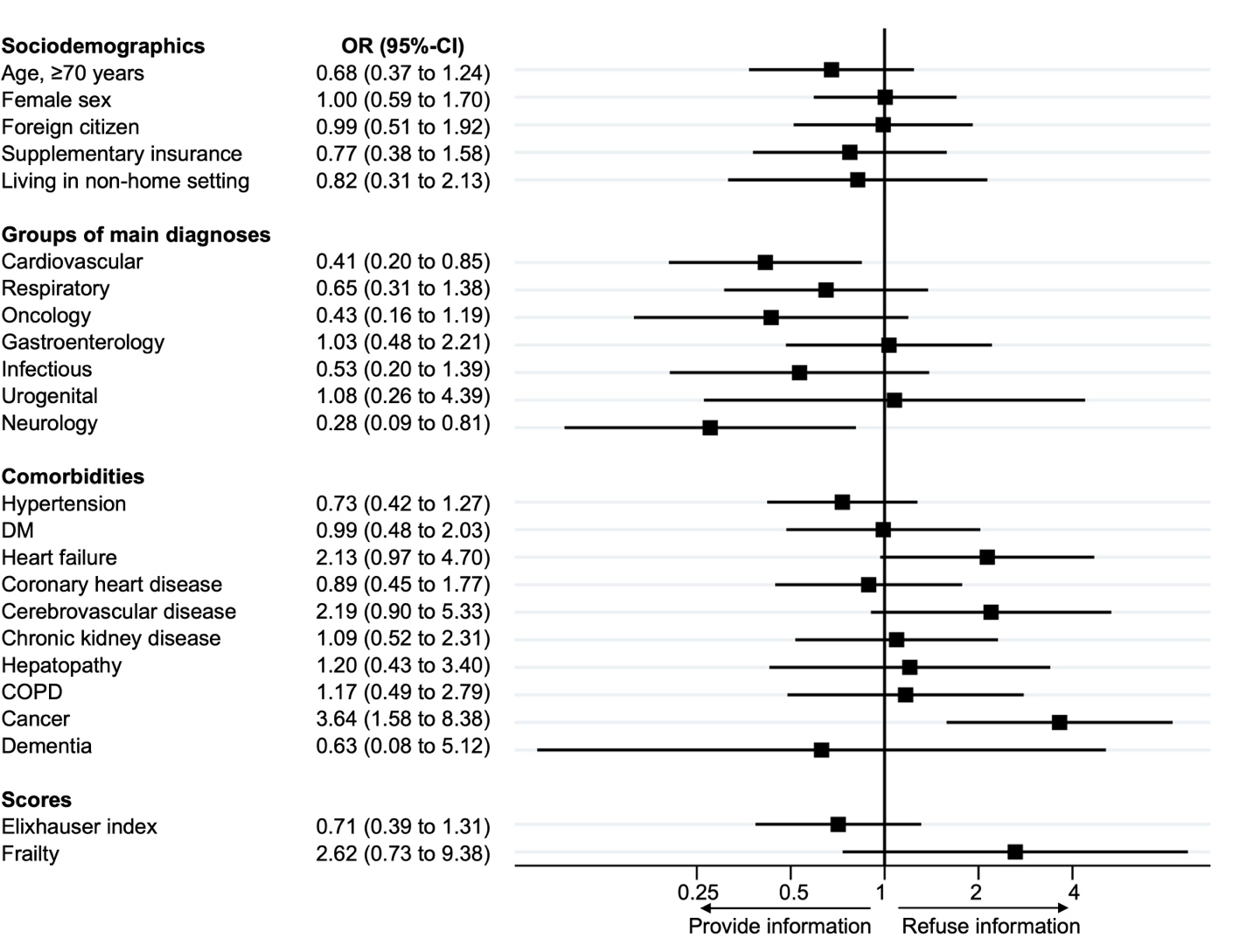


The forest plot depicts graphical association between patient determinants with people who were willing to provide information and those who refused after the first hospitalization in the study period only. Determinants are on the y-axis and odds ratio on the x-axis.

Model specification: Mixed-effects logistic regression with patient-specific random intercepts

# **Table S1 Baseline characteristics of responders and non-responders after the first hospitalization in the study period only**

|  | **Responder** | **Non-Responder** | **P-value** | **SMD** |
| --- | --- | --- | --- | --- |
| **N** | N=20,252 | N=3,941 |  |  |
| **Patient Characteristics** |  |  |  |  |
| Age, median (IQR) | 72.0 (60.0-82.0) | 71.0 (56.0-83.0) | 0.038 | -0.07 |
| Female, n (%) | 9,773 (48.3) | 1,943 (49.3) | 0.23 | 0.02 |
| Swiss citizen, n (%) | 16,643 (82.2) | 2,950 (74.9) | <0.001 | -0.18 |
| Insurance |  |  | <0.001 | 0.12 |
| Basic, n (%) | 15,644 (77.2) | 3,237 (82.1) |  |  |
| Supplementary, n (%) | 4,608 (22.7) | 704 (17.8) |  |  |
| Admission |  |  | <0.001 | 0.19 |
| from home, n (%) | 18,239 (90.1) | 3,308 (83.9) |  |  |
| from facility, n (%) | 1,602 ( 7.9) | 543 (13.8) |  |  |
| other, n (%) | 411 ( 2.0) | 90 ( 2.3) |  |  |
| **Comorbidities** |  |  |  |  |
| Cardiovascular, n (%) | 6,147 (30.4) | 931 (23.6) | <0.001 | -0.15 |
| Respiratory, n (%) | 2,828 (14.0) | 532 (13.5) | 0.44 | -0.01 |
| Oncology, n (%) | 877 ( 4.3) | 361 ( 9.2) | <0.001 | 0.19 |
| Gastroenterology, n (%) | 1,728 ( 8.5) | 357 ( 9.1) | 0.28 | 0.02 |
| Infectious, n (%) | 1,525 ( 7.5) | 296 ( 7.5) | 0.97 | 0.00 |
| Urogenital, n (%) | 829 ( 4.1) | 193 ( 4.9) | 0.022 | 0.04 |
| Neurology, n (%) | 1,732 ( 8.6) | 274 ( 7.0) | <0.001 | -0.06 |
| Hypertension, n (%) | 11,327 (55.9) | 2,002 (50.8) | <0.001 | -0.10 |
| DM, n (%) | 4,031 (19.9) | 780 (19.8) | 0.87 | 0.00 |
| Heart failure, n (%) | 2,726 (13.5) | 535 (13.6) | 0.85 | 0.00 |
| Coronary heart disease, n (%) | 4,933 (24.4) | 806 (20.5) | <0.001 | -0.09 |
| Cerebrovascular disease, n (%) | 2,831 (14.0) | 520 (13.2) | 0.19 | -0.02 |
| Chronic kidney disease, n (%) | 4,222 (20.8) | 878 (22.3) | 0.044 | 0.03 |
| Hepatopathy, n (%) | 702 ( 3.5) | 202 ( 5.1) | <0.001 | 0.08 |
| COPD, n (%) | 1,797 ( 8.9) | 346 ( 8.8) | 0.85 | 0.00 |
| Cancer, n (%) | 1,853 ( 9.1) | 575 (14.6) | <0.001 | 0.17 |
| Dementia, n (%) | 1,098 ( 5.4) | 322 ( 8.2) | <0.001 | 0.11 |
| Elixhauser Comorbidity Index^a^, mean (SD) | 2.8 (2.0) | 3.1 (2.0) | <0.001 | 0.16 |
| Frailty Risk Score^b^, n (%) |  |  | <0.001 | 0.17 |
| low | 13,840 (68.3) | 2,393 (60.7) |  |  |
| intermediate | 5,808 (28.7) | 1,359 (34.5) |  |  |
| high | 604 ( 3.0) | 189 ( 4.8) |  |  |

SMD, standardized mean difference; IQR, interquartile range; SD standard deviation; DM, diabetes mellitus; COPD, chronic obstructive pulmonary disease.

^a^ Scores range from -7 to 12 with higher scores indicating greater comorbidity.

^b^ Scores range from 0 to 99 with higher scores indicating greater frailty.

# **Table S2 Baseline characteristics of responders and non-responders with discharge to home**

|  | **Responder** | **Non-Responder** | **P-value** | **SMD** |
| --- | --- | --- | --- | --- |
| **N** | N=19,260 | N=3,372 |  |  |
| **Patient Characteristics** |  |  |  |  |
| Age, median (IQR) | 71.0 (58.0-80.0) | 65.0 (53.0-77.0) | <0.001 | -0.24 |
| Female, n (%) | 8,933 (46.4) | 1,575 (46.7) | 0.73 | 0.01 |
| Swiss citizen, n (%) | 15,431 (80.1) | 2,287 (67.8) | <0.001 | -0.28 |
| Insurance |  |  | <0.001 | 0.16 |
| Basic, n (%) | 14,858 (77.1) | 2,816 (83.5) |  |  |
| Supplementary, n (%) | 5,840 (22.9) | 993 (17.9) |  |  |
| Admission |  |  | 0.49 | 0.02 |
| from home, n (%) | 18,753 (97.4) | 3,285 (97.4) |  |  |
| from facility, n (%) | 310 ( 1.6) | 59 ( 1.7) |  |  |
| other, n (%) | 197 ( 1.0) | 28 ( 0.8) |  |  |
| Number of hospitalizations |  |  | <0.001 | 0.21 |
| 1, n (%) | 15,322 (79.6) | 2,392 (70.9) |  |  |
| 2, n (%) | 3,308 (17.2) | 775 (23.0) |  |  |
| ≥3, n (%) | 630 ( 3.3) | 205 ( 6.1) |  |  |
| **Comorbidities** |  |  |  |  |
| Cardiovascular, n (%) | 5,304 (27.5) | 653 (19.4) | <0.001 | -0.19 |
| Respiratory, n (%) | 2,852 (14.8) | 498 (14.8) | 0.95 | 0.00 |
| Oncology, n (%) | 927 ( 4.8) | 356 (10.6) | <0.001 | 0.22 |
| Gastroenterology, n (%) | 1,868 ( 9.7) | 369 (10.9) | 0.026 | 0.04 |
| Infectious, n (%) | 1,545 ( 8.0) | 265 ( 7.9) | 0.75 | -0.01 |
| Urogenital, n (%) | 808 ( 4.2) | 159 ( 4.7) | 0.17 | 0.03 |
| Neurology, n (%) | 1,614 ( 8.4) | 236 ( 7.0) | 0.007 | -0.05 |
| Hypertension, n (%) | 10,439 (54.2) | 1,552 (46.0) | <0.001 | -0.16 |
| DM, n (%) | 3,785 (19.7) | 686 (20.3) | 0.35 | 0.02 |
| Heart failure, n (%) | 2,369 (12.3) | 366 (10.9) | 0.017 | -0.05 |
| Coronary heart disease, n (%) | 4,707 (24.4) | 689 (20.4) | <0.001 | -0.10 |
| Cerebrovascular disease, n (%) | 2,012 (10.4) | 250 ( 7.4) | <0.001 | -0.11 |
| Chronic kidney disease, n (%) | 3,879 (20.1) | 658 (19.5) | 0.40 | -0.02 |
| Hepatopathy, n (%) | 717 ( 3.7) | 184 ( 5.5) | <0.001 | 0.08 |
| COPD, n (%) | 1,751 ( 9.1) | 327 ( 9.7) | 0.26 | 0.02 |
| Cancer, n (%) | 2,018 (10.5) | 570 (16.9) | <0.001 | 0.19 |
| Dementia, n (%) | 642 ( 3.3) | 128 ( 3.8) | 0.17 | 0.02 |
| Elixhauser Comorbidity Index^a^, mean (SD) | 2.7 (1.9) | 2.8 (2.0) | <0.001 | 0.09 |
| Frailty Risk Score^b^, n (%) |  |  | 0.013 | 0.05 |
| low | 14,546 (75.5) | 2,488 (73.8) |  |  |
| intermediate | 4,452 (23.1) | 820 (24.3) |  |  |
| high | 262 ( 1.4) | 64 ( 1.9) |  |  |

SMD, standardized mean difference; IQR, interquartile range; SD standard deviation; DM, diabetes mellitus; COPD, chronic obstructive pulmonary disease.

^a^ Scores range from -7 to 12 with higher scores indicating greater comorbidity.

^b^ Scores range from 0 to 99 with higher scores indicating greater frailty.

# **Table S3 Baseline characteristics of responders and non-responders with discharge to non-home setting**

|  | **Responder** | **Non-Responder** | **P-value** | **SMD** |
| --- | --- | --- | --- | --- |
| **N** | N=6,297 | N=2,173 |  |  |
| **Patient Characteristics** |  |  |  |  |
| Age, median (IQR) | 79.0 (68.0-85.0) | 80.0 (68.0-86.0) | 0.070 | 0.00 |
| Female, n (%) | 3,288 (52.2) | 1,147 (52.8) | 0.65 | 0.01 |
| Swiss citizen, n (%) | 5,512 (87.5) | 1,838 (84.6) | <0.001 | -0.09 |
| Insurance |  |  | 0.027 | 0.07 |
| Basic, n (%) | 4,859 (77.2) | 1,736 (79.9) |  |  |
| Supplementary, n (%) | 5,840 (22.9) | 993 (17.9) |  |  |
| Admission |  |  | <0.001 | 0.10 |
| from home, n (%) | 4,196 (66.6) | 1,364 (62.8) |  |  |
| from facility, n (%) | 1,811 (28.8) | 720 (33.1) |  |  |
| other, n (%) | 290 ( 4.6) | 89 ( 4.1) |  |  |
| Number of hospitalizations |  |  | <0.001 | 0.18 |
| 1, n (%) | 4,930 (78.3) | 1,549 (71.3) |  |  |
| 2, n (%) | 1,132 (18.0) | 475 (21.9) |  |  |
| ≥3, n (%) | 235 ( 3.7) | 149 ( 6.9) |  |  |
| **Comorbidities** |  |  |  |  |
| Cardiovascular, n (%) | 2,294 (36.4) | 640 (29.5) | <0.001 | -0.15 |
| Respiratory, n (%) | 854 (13.6) | 276 (12.7) | 0.31 | -0.03 |
| Oncology, n (%) | 229 ( 3.6) | 206 ( 9.5) | <0.001 | 0.24 |
| Gastroenterology, n (%) | 375 ( 6.0) | 165 ( 7.6) | 0.007 | 0.07 |
| Infectious, n (%) | 376 ( 6.0) | 147 ( 6.8) | 0.19 | 0.03 |
| Urogenital, n (%) | 270 ( 4.3) | 86 ( 4.0) | 0.51 | -0.02 |
| Neurology, n (%) | 525 ( 8.3) | 150 ( 6.9) | 0.033 | -0.05 |
| Hypertension, n (%) | 4,013 (63.7) | 1,281 (59.0) | <0.001 | -0.10 |
| DM, n (%) | 1,511 (24.0) | 495 (22.8) | 0.25 | -0.03 |
| Heart failure, n (%) | 1,286 (20.4) | 434 (20.0) | 0.65 | -0.01 |
| Coronary heart disease, n (%) | 1,762 (28.0) | 496 (22.8) | <0.001 | -0.12 |
| Cerebrovascular disease, n (%) | 1,569 (24.9) | 480 (22.1) | 0.008 | -0.07 |
| Chronic kidney disease, n (%) | 1,884 (29.9) | 675 (31.1) | 0.32 | 0.02 |
| Hepatopathy, n (%) | 249 ( 4.0) | 120 ( 5.5) | 0.002 | 0.07 |
| COPD, n (%) | 777 (12.3) | 221 (10.2) | 0.007 | -0.07 |
| Cancer, n (%) | 506 ( 8.0) | 345 (15.9) | <0.001 | 0.24 |
| Dementia, n (%) | 774 (12.3) | 312 (14.4) | 0.013 | 0.06 |
| Elixhauser Comorbidity Index^a^, mean (SD) | 3.7 (2.0) | 3.9 (2.0) | <0.001 | 0.11 |
| Frailty Risk Score^b^, n (%) |  |  | <0.001 | 0.13 |
| low | 2,689 (42.7) | 790 (36.4) |  |  |
| intermediate | 3,061 (48.6) | 1,158 (53.3) |  |  |
| high | 547 ( 8.7) | 225 (10.4) |  |  |

SMD, standardized mean difference; IQR, interquartile range; SD standard deviation; DM, diabetes mellitus; COPD, chronic obstructive pulmonary disease.

^a^ Scores range from -7 to 12 with higher scores indicating greater comorbidity.

^b^ Scores range from 0 to 99 with higher scores indicating greater frailty.
