# Supplementary material for: Determinants associated with completion of postdischarge follow-up survey among multimorbid patients: a secondary analysis of the non-randomised clinical In-HospiTOOL trial
Source: BMJ Open. 2025 Dec 12;15(12):e105210. doi: 10.1136/bmjopen-2025-105210 (PMC12706230; doi:10.1136/bmjopen-2025-105210)
Supplement: online supplemental file 2 [file bmjopen-15-12-s002.pdf]

In-HospiTOOL_Interview 30 Tage nach Eintritt
(Version 13.7.2017)

Secutrial Information:
Datum
Clinical Investigator
Projekt
Zentrum
Patient Zus-ID
Tag 30 Datum tt.mm.jjjj
Formularfamilie
Formular

Interview
Klinik, OE
Patientenummer

Patient
Aufnahmedatum Datum tt.mm.jjjj
Austrittsdatum Datum tt.mm.jjjj
Austrittsort
Name
Vorname
Adresse
Adresszusatz
PLZ
Ort
Telefon 1
Telefon 2
Telefon 3
Telefon 4
E-Mail
Geschlecht Männlich, weiblich
Geburtsdatum Datum tt.mm.jjjj

Bezugspersonen
Name
Telefon 1
Telefon 2
Hausarzt
Name
Vorname
Ort
Telefon
Bemerkungen

Interviewer
Interviewdatum Datum tt.mm.jjjj
Initialen Interviewer

1. Erreichbarkeit
1.1. Interview hat stattgefunden Ja Nein
Falls "Ja", erreicht am: Datum tt.mm.jjjj
Falls "Nein": Grund
- Patient ist verstorben
- Patient will keine Auskunft geben
- Patient nicht erreicht
- Fremdsprache

- anderer

1.2. Interview wird geführt mit
- Patient selbst
- Lebenspartner
- Betreuende Person (Pflegende, Betreuer, Beistand, etc.)
- Familienmitglied
- Bezugsperson (z.B. Nachbar, Bekannte Kollege, etc.)
- Andere

Falls Interview nicht mit Patient selber durchgeführt, bitte Grund angeben: (nur 1 Grund auswählbar)
- Fremdsprache
- Aphasie
- Schwerhörigkeit
- kognitive Einschränkung (dement, desorientiert, behindert)
- terminaler Patient
- komatöser/intubierter Patient
- ist zu schwach/krank um Auskunft zu geben
- ist im Ausland
- anderes: -> Stichwort

2. Fragen zum Verlauf nach der Spital Entlassung
2.1. Mussten Sie seit dem Spitalaustritt ungeplant/unvohergesehen/notfallmässig wieder 1 oder mehrere Nächte im Spital verbringen? Ja Nein
A. Datum der 1. (Re)Hospitalisation: Datum tt.mm.jjjj
B. Grund? gleiches Problem anderes Problem unbekannt

Falls ja:
C. Im gleichen Spital? Ja Nein unbekannt
D. Wurde Notfalleingriff/Notfalloperation nötig? Ja Nein unbekannt
(Zusatzinfo-Box: Lungenspiegelung, Röntgen, Magen-Darm-Spiegelung, Herzkatheter-Untersuchung, Stent, etc.)

2.2. Mussten Sie seit dem Spitalaustritt ungeplant/unvohergesehen/notfallmässig auf die Notfallstation/in die NF-Praxis gehen? Ja Nein
A. Datum der 1. Besuchs der Notfallstation/-praxis: Datum tt.mm.jjjj
B. Grund? gleiches Problem anderes Problem unbekannt

Falls ja:
C. Im gleichen Spital? Ja Nein unbekannt

2.3. Mussten Sie seit dem Spitalaustritt ungeplant/unvohergesehen/notfallmässig zu Ihrem Hausarzt gehen?
Ja Nein
Wie oft?
A. Datum der 1. ungeplanten Besuchs beim Hausarzt: Datum tt.mm.jjjj
B. Grund? gleiches Problem anderes Problem unbekannt

3. Fragen zur Zufriedenheit mit Spitalaufenthalt
3.1 Wie zufrieden waren Sie mit dem Aufenthalt auf der Notfallstation vor 30 Tagen auf einer Skala von 0-10?
(0= sehr unzufrieden; 5=neutral; 10=sehr zufrieden)
Unbekannt

Wenn <8 weshalb? (Mehrfachauswahl möglich):
- Wartezeiten
- Essen/Trinken
- Harte Liege
- AD-Kommunikation/Information (Höflichkeit, fehlende/späte Infos etc.)
- PD-Kommunikation/Information (Höflichkeit, fehlende/späte Infos etc.)
- AD-Arbeitsqualität/Fachkompetenz

- PD-Arbeitsqualität/Fachkompetenz
- andere Dienste: Arbeitsqualität/Fachkompetenz
- Patienten-Aufnahme: Arbeitsqualität/Fachkompetenz
- anderes *(-> Freitextfeld)* ->Stichwort

Erläuterungen zur Unzufriedenheit: ->Stichworte: *(-> Freitextfeld)*

(Keine konkreten Gründe aus der Liste vorschlagen (Beeinflussung!). Falls nötig übergeordnet Fragen „Was hat Sie am meisten gestört? Was hat Ihnen gefehlt?“)

3.2 Wie zufrieden sind sie generell mit dem Spitalaufenthalt vor 30 Tagen auf einer Skala von 0-10? Zahl 0-10 (0= sehr unzufrieden; 5=neutral; 10=sehr zufrieden)
Unbekannt

3.2.1 Was haben Sie am Aufenthalt am meisten geschätzt? (Mehrfachauswahl möglich):
- AD-Kommunikation/Information (Höflichkeit, passende und zeitgerechte Infos, persönliche Anliegen wahrgenommen, etc.)
- PD-Kommunikation/Information (Höflichkeit, passende und zeitgerechte Infos, persönliche Anliegen wahrgenommen, etc.)
- AD-Arbeitsqualität/Fachkompetenz
- PD-Arbeitsqualität/Fachkompetenz
- andere Dienste Arbeitsqualität/Fachkompetenz
- Zimmer/Bett/Bad/Einrichtung
- Essen/Trinken
- Hygiene/Reinigung
- kurze Wartezeiten
- Zimmernachbar/Besuch
- anderes -> Stichwort: *(-> Freitextfeld)*
- keine positive Rückmeldung

Erläuterungen zur Zufriedenheit: ->Stichworte: *(-> Freitextfeld)*

(Keine konkreten Gründe aus der Liste vorschlagen (Beeinflussung)!. Falls nötig übergeordnet Fragen „Gab es vielleicht einen Dienst, welchen Sie besonders geschätzt haben? …etc.)

3.2.2 Was haben sie am Aufenthalt am wenigsten geschätzt? (Mehrfachauswahl möglich):
- AD-Kommunikation/Information (Höflichkeit, fehlende/späte Infos etc.)
- PD-Kommunikation/Information (Höflichkeit, fehlende/späte Infos etc.)
- AD-Arbeitsqualität/Fachkompetenz
- PD-Arbeitsqualität/Fachkompetenz
- andere Dienste Arbeitsqualität/Fachkompetenz
- Mängel in der interdisziplinären Kommunikation (bspw. Zu viele unterschiedliche Informationen von verschiedenen Seiten)
- Zimmer/Bett/Bad/Einrichtung
- Essen/Trinken
- Hygiene/Reinigung
- lange Wartezeiten
- Zimmernachbar/Besuch
- anderes -> Stichwort: *(-> Freitextfeld)*
- keine negative Rückmeldung

Erläuterungen zur Unzufriedenheit: ->Stichworte: *(-> Freitextfeld)*

(Keine konkreten Gründe aus der Liste vorschlagen (Beeinflussung!). Falls nötig übergeordnet Fragen „Gab es vielleicht einen Dienst, welchen Sie nicht geschätzt haben? …etc.)

4. Zufriedenheit mit dem Austrittsprozess

4.1. Fühlten Sie sich über Diagnose und Behandlung gut informiert? Zahl 0-10 (0=nein, gar nicht; 10=ja, sehr)
Unbekannt

4.2 Wurden Sie in die Austrittsplanung einbezogen? Zahl 0-10 (0 = gar nicht; 10= vollständig)
war nicht nötig unbekannt

4.3 „Wurden Sie im Spital gebeten die Informationen vom Arzt- und Pflegedienst, die sie erhalten haben in eigenen Worten zu wiederholen?“
(Mussten Sie z.B. in eigenen Worten beschreiben, wie Sie zu Hause damit umgehen, wenn die Symptome, die zu Ihrem Spitaleintritt geführt hatten wieder auftreten?)

Arzt: Ja Nein unbekannt
Pflege: Ja Nein unbekannt

4.4 War vom Spital für das Leben zuhause bzw. die weitere Nachbetreuung alles gut vorbereitet worden (z.B. Medikament, Hilfsmittel)?
Ja, eindeutig eher schon eher nicht Nein, überhaupt nicht es braucht keine Vorbereitung Unbekannt
Falls "eher nicht" bzw. "Nein, überhaupt nicht" Stichwort

(Info: z. Bsp: Termine bekannt, Weiterführung der Therapie, Spitex/Nachbetreuung geplant & kommuniziert, Austrittsdokumente erhalten, Hilfsmittel, O2-Th., Angehörige informiert)

Grund: drop down Auswahlliste *(Mehrfachauswahl):*
AD - kein Austrittsgespräch
AD - Austrittsunterlagen nicht abgegeben (Austrittsbericht, Rezept, Verordnungen, Termine, etc.)
AD – Austrittsunterlagen fehlerhaft
AD – fehlende Austrittsinformationen/Instruktionen (Austrittsprocedere, Krankheitsverlauf, Aufklärung, Verhaltensregeln, Prophylaxe)
AD – fehlende Angehörigeninformation
PD – Austrittsunterlagen nicht abgegeben (Austrittsbericht, eigene Medikamente und Materialien, Ausweise, etc. nicht zurückgegeben
PD – Austrittsunterlagen fehlerhaft (zu wenig Medikamente, Verbandsmaterial, etc.)
PD – fehlende Austrittsinformationen/Instruktionen (Austrittsprocedere, Verhaltensregeln, Prophylaxe, zurechtfinden im Alltag, etc.)
PD – fehlende Angehörigeninformation
Ungenügende Medikamenteninformation, Instruktion
Nachbetreuung nicht organisiert (Spitex Pflege und Haushalt, etc.)
Austrittsort entspricht nicht dem Patientenwunsch
Hilfsmittel fehlten: (Rollator, Rollstuhl, etc.)
Mängel in der interdisziplinären Kommunikation (bspw. zu viele unterschiedliche Informationen von verschiedenen Seiten)
andere: ->Stichwort
Erläuterungen zur Unzufriedenheit: ->Stichworte: (-> Freitextfeld)

(Keine konkreten Gründe aus der Liste vorschlagen (Beeinflussung!). Falls nötig übergeordnet Fragen „Hat Ihnen etwas für den Austritt gefehlt oder haben Sie Informationen vermisst?“…etc.)

<u>5. Fragen zur Lebensqualität</u>
5.1 Welche der folgenden Aussagen beschreibt ihren Gesundheitszustand (aktuell und vor Spitaleintritt (Zeitraum 4 Wochen) am besten?
(5.1.1_5.1.2, etc. bis 5.6.1_ 5.6.2)

a) aktuell, 30d nach Eintritt
b) Vor Spitaleintritt (Zeitraum letzte 4 Wochen) = innerhalb der letzten 4 Wochen vor Spitaleintritt
Bei jeder Frage unbekannt aktuell und vor Spitaleintritt (letzte 4 Wochen)

Beweglichkeit/Mobilität
- Ich habe keine Probleme herumzugehen
- Ich habe leichte Probleme herumzugehen

- Ich habe mässige Probleme herumzugehen
- Ich habe grosse Probleme herumzugehen
- Ich bin nicht in der Lage herumzugehen

Für sich selber sorgen
- Ich habe keine Probleme, mich selbst zu waschen oder anzuziehen
- Ich habe leichte Probleme, mich selbst zu waschen oder anzuziehen
- Ich habe mässige Probleme, mich selbst zu waschen oder anzuziehen
- Ich habe grosse Probleme, mich selbst zu waschen oder anzuziehen
- Ich bin nicht in der Lage, mich selbst zu waschen oder anzuziehen

Alltägliche Tätigkeiten (z.B. Arbeit, Studium, Hausarbeit, Familien- oder Freizeitaktivitäten)
- Ich habe keine Probleme, meinen alltäglichen Tätigkeiten nachzugehen
- Ich habe leichte Probleme, meinen alltäglichen Tätigkeiten nachzugehen
- Ich habe mässige Probleme, meinen alltäglichen Tätigkeiten nachzugehen
- Ich habe grosse Probleme, meinen alltäglichen Tätigkeiten nachzugehen
- Ich bin nicht in der Lage, meinen alltäglichen Tätigkeiten nachzugehen

Schmerzen/körperliche Beschwerden
- Ich habe keine Schmerzen oder Beschwerden
- Ich habe leichte Schmerzen oder Beschwerden
- Ich habe mässige Schmerzen oder Beschwerden
- Ich habe starke Schmerzen oder Beschwerden
- Ich habe extreme Schmerzen oder Beschwerden

Angst/Niedergeschlagenheit
- Ich bin nicht ängstlich oder deprimiert
- Ich bin ein wenig ängstlich oder deprimiert
- Ich bin mässig ängstlich oder deprimiert
- Ich bin sehr ängstlich oder deprimiert
- Ich bin extrem ängstlich oder deprimiert

a) Wie gut oder wie schlecht schätzen Sie Ihren Gesundheitszustand auf einer Skala von 0-100% heute ein (ähnlich einem Thermometer)
% (0=der schlechteste Gesundheitszustand; 100=der best denkbare Gesundheitszustand)
unbekannt

b) Wie gut oder wie schlecht schätzen Sie Ihren Gesundheitszustand auf einer Skala von 0-100% vor Spitaleintritt (Zeitraum 4 Wochen) ein (ähnlich einem Thermometer)
% (0=der schlechteste Gesundheitszustand; 100=der best denkbare Gesundheitszustand)
unbekannt

5.7 Benötigen Sie aktuell Unterstützung bei:

1) Medikamenteneinnahme und/oder Bereitstellung	Ja	Nein	unbekannt
2) Essen/Trinken	Ja	Nein	unbekannt
3) Ausscheidung	Ja	Nein	unbekannt

5.8 Benötigten Sie vor der Spitaleintritt (Zeitraum 4 Wochen) Unterstützung bei:

1) Medikamenteneinnahme und/oder Bereitstellung	Ja	Nein	unbekannt
2) Essen/Trinken	Ja	Nein	unbekannt
3) Ausscheidung	Ja	Nein	unbekannt

6. Fragen zur Veränderung der Wohn und Betreuungssituation

Hat sich Ihre aktuelle Wohnsituation im Vergleich zu von dem Spitalaufenthalt verändert?

Ja Nein unbekannt

6.1

- Neu in Alters-, Pflegeheim
- Noch im Spital
- Wieder im Spital
- Wohnt neu bei Familie/Bezugsperson
- Noch in Akutgeriatrie, Übergangspflege
- Noch in Rehabilitation/Kur
- Noch in Ferienbett
- Neu betreutes Wohnen
- Neu Hospiz/Palliative Care
- Anderes: -> Stichwort

6.2 Betreuungssituation aktuell (30d nach Spitaleintritt):

Brauchen Sie zurzeit Hilfe im Alltag? Ja Nein unbekannt

Wenn Ja, wer leistet die Hilfe:

- Institution (Reha, Spital, Klinik, Pflegeheim, etc.)
- Spitex Pflege oder äquivalentes Angebot
- Spitex Haushalt oder äquivalentes Angebot
- Angehörige/Bezugsperson
- Mahlzeitendienst
- Transportdienst
- andere

6.3 Betreuungssituation vor Spitaleintritt (innerhalb der letzten 4 Wochen)

Brauchten Sie vor Ihrem Spitaleintritt Hilfe im Alltag? Ja Nein unbekannt

Wenn Ja, wer leistete die Hilfe: *(Mehrfachauswahl)*

- Institution (Reha, Spital, Klinik, Pflegeheim, etc.)
- Spitex Pflege oder äquivalentes Angebot
- Spitex Haushalt oder äquivalentes Angebot
- Angehörige/Bezugsperson
- Mahlzeitendienst
- Transportdienst
- andere

7. Es folgen nun einige Fragen welche uns helfen Ihren Gesundheitszustand besser einzuschätzen.

7.1 Haben Sie aktuell Probleme Urin zurückhalten zu können (Inkontinenz)?

Ja Nein unbekannt

7.1.1 Wenn ja (im Vergleich zu vor Spitaleintritt [4 Wochen]):
Neu aufgetreten
Weniger als vor Spitaleintritt
Mehr als vor Spitaleintritt
Gleich

7.1.2 Wenn nein Vor Spitaleintritt kein Problem (Zeitraum 4 Wochen)
Vor Spitaleintritt Problem (Zeitraum 4 Wochen)

7.2 Haben Sie aktuell Probleme Stuhlgang zurückhalten zu können (Inkontinenz)?
Ja Nein unbekannt

7.2.1 Wenn ja (im Vergleich zu vor Spitaleintritt [Zeitraum 4 Wochen]):
Neu aufgetreten
Weniger als vor Spitaleintritt
Mehr als vor Spitaleintritt
Gleich

7.2.2 Wenn nein Vor Spitaleintritt kein Problem (Zeitraum 4 Wochen)
Vor Spitaleintritt Problem (Zeitraum 4 Wochen)

7.3 Leiden Sie aktuell unter Durchfall?
Ja Nein unbekannt

7.3.1 Wenn ja (im Vergleich zu vor Spitaleintritt [Zeitraum 4 Wochen]):
Neu aufgetreten
Weniger als vor der Hosp.
Mehr als vor der Hosp.
Gleich

7.3.2 Wenn nein: Vor Spitaleintritt kein Problem (Zeitraum 4 Wochen)
Vor Spitaleintritt Problem (Zeitraum 4 Wochen)

7.4 Haben Sie seit dem Spitalaustritt an Gewicht verloren?
Ja Nein unbekannt

7.5 Appetit
Wie ist ihr Appetit auf einer Skala von 0-10? Zahl 0-10 *(0=überhaupt kein Appetit; 10=bester Appetit mit keinerlei Einschränkungen)*

7.5.1
- Weniger als vor Spitaleintritt (Zeitraum 4 Wochen)
- Mehr als vor Spitaleintritt (Zeitraum 4 Wochen)
- Gleich
- unbekannt

7.6. Sind Sie seit der Spitalentlassung gestürzt ? Ja Nein unbekannt
7.6.1 Wenn ja: einmal mehrmals unbekannt
7.6.2 Haben Sie sich dabei einen Knochenbruch zugezogen? Ja Nein unbekannt

7.6.3 Sind Sie vor dem Spitaleintritt gestürzt (innerhalb der letzten 4 Wochen)? Ja Nein unbekannt
7.6.4 Wenn ja: einmal mehrmals unbekannt
7.6.5 Haben Sie sich dabei einen Knochenbruch zugezogen? Ja Nein unbekannt

7.7 Leiden Sie aktuell unter Schlafstörungen (Probleme mit Ein- und/oder Durchschlafen)?

Ja Nein unbekannt

7.7.1 Wenn ja (im Vergleich zu vor Spitaleintritt [Zeitraum 4 Wochen]):
Neu aufgetreten
Weniger als vor der Hospitalisation
Mehr als vor der Hospitalisation
Gleich
unbekannt

7.7.2 Wenn nein: Vor Spiteintritt (4Wochen) kein Problem
Vor Spitaleintritt Problem
unbekannt

7.8 Leiden Sie aktuell unter einer starken, ungewöhnlichen Müdigkeit/Erschöpfung?
(Energie reicht trotz guter Nachtruhe nicht für den ganzen Tag – „Akku“ kann nicht mehr genügend geladen werden, massiv erhöhtes Ruhebedürfnis, das absolut unverhältnismäßig zu vorausgegangenen Aktivitäten ist)
Ja Nein unbekannt

7.8.1 Wenn ja (im Vergleich zu vor Spitaleintritt [Zeitraum 4 Wochen]):
neu aufgetreten
Weniger als vor der Hospitalisation
Mehr als vor der Hospitalisation
Gleich
unbekannt

7.8.2 Wenn nein: Keine ungewöhnliche Müdigkeit vor Spitaleintritt (Zeitraum 4 Wochen)
Ungewöhnliche Müdigkeit vor Spitaleintritt vorhanden
unbekannt

7.9 Haben Sie eine Blutzuckerkrankheit (Diabetes)?
Ja Nein unbekannt

7.9.1 wenn Ja:
-> Empfinden Sie die Einstellung der Blutzuckerwerte im Vergleich zu vor der Hospitalisation (Zeitraum 4 Wochen) bezüglich - Unterzuckerung (Hypo) oder - Überzuckerung (Hyper) als :
besser gleich schlechter unbekannt

7.10 Leiden Sie aktuell unter Atemnot?
Ja Nein unbekannt

7.10.1 wenn Ja:
a) Klassifikation gemäss NYHA I-IV;
I= keine Einschränkung im Alltag
II=leichte Einschränkung im Alltag – Atemnot bei Treppensteigen zwei Stockwerke (=bei stärkerer Belastung)
III=höhergradige Einschränkung im Alltag –Atemnot bei Verrichtungen im Haushalt (=bei leichter Belastung)
IV=hochgradige Einschränkung im Alltag - auch in Ruhe
Unbekannt)

7.10.2 Wie stark war die Atemnot vor dem Spitaleintritt (Zeitraum 4 Wochen)?
Keine Atemnot vor Spitaleintritt
I= keine Einschränkung im Alltag
II=leichte Einschränkung im Alltag – Atemnot bei Treppensteigen zwei Stockwerke (=bei stärkerer Belastung)

III=höhergradige Einschränkung im Alltag –Atemnot bei Verrichtungen im Haushalt (=bei leichter Belastung)
IV=hochgradige Einschränkung im Alltag - auch in Ruhe
Unbekannt)

7.11 Sind Sie Raucher? (alle Formen von Tabakkonsum, mit und ohne Lungeninhalation)
(Inklusive: Zigaretten, Pfeife, Stumpen, Zigarren, E-Zigaretten etc.)
- Ja
- Nein, nie geraucht
- Nein (schon vor Spitaleintritt gestoppt)
- Nein (Stopp seit Spitalaufenthalt)
- unbekannt

Falls Ja:
Menge aktuell (im Vergleich letzte 4 Wochen vor Spitaleintritt)
Mehr gleich viel weniger unbekannt
Rauchstopp besprochen? Ja Nein unbekannt

Falls Nein, Stopp seit Spitalaufenthalt:
Rauchstopp besprochen? Ja Nein unbekannt

7.12 Blutungskomplikationen?
Hatten Sie seit der Spitalentlassung eine Blutung? (z.B.. Nasenbluten, Bluterguss, Blut im Stuhl, Sputum etc).
Nein Ja unbekannt

8 Fragen zur Erwerbstätigkeit
8.1 Sind sie zurzeit wieder erwerbstätig?
- Nein, ich habe bereits vor der Hospitalisation nicht gearbeitet
- Nein, ich habe seit der Hospitalisation nicht gearbeitet
- Ja
- unbekannt

Falls "ja":
Zu wieviel %? %
Wieviel % betrug ihre vorherige Tätigkeit? %
Seit wann arbeiten Sie wieder? Datum tt.mm.jjjj
Unbekannt

9 Kommentare
Freitext
