# Supplementary material for: Determinants associated with completion of postdischarge follow-up survey among multimorbid patients: a secondary analysis of the non-randomised clinical In-HospiTOOL trial
Source: BMJ Open. 2025 Dec 12;15(12):e105210. doi: 10.1136/bmjopen-2025-105210 (PMC12706230; doi:10.1136/bmjopen-2025-105210)
Supplement: Supplementary file 3 [file bmjopen-2025-105210.reviewer_comments.pdf]

PEER REVIEW HISTORY

BMJ Open publishes all reviews undertaken for accepted manuscripts. Reviewers are asked to complete a checklist review form and are provided with free text boxes to elaborate on their assessment. These free text comments are reproduced below.

ARTICLE DETAILS

Title (Provisional)

Determinants associated with completion of postdischarge follow-up survey among multimorbid patients – a secondary analysis of the nonrandomized clinical In-HospiTOOL trial

Authors

Thuraisingam, Hagena; Laager, Rahel; Gregoriano, Claudia; Schuetz, Philipp; Mueller, Beat; Kutz, Alexander

---

VERSION 1 - REVIEW

**Reviewer**	**1**
**Name**	**Ang, Ian**
**Affiliation**	**National University Singapore Saw Swee Hock School of Public Health**
**Date**	**28-Jul-2025**
**COI**	**None**

This study is a secondary analysis of data collected as part of the In-HospiTOOL trial, to assess the factors that are associated with completion of PREMs and PROMs through a phone survey. PREMs and PROMs are important for monitoring and improvement of patient management and care, and so understanding the factors that influence the data collection process is important. The authors provided a strong rationale for this study for which their approach for the most part contain the relevant parts to fulfil the study objectives/research aims. However, there are some potential major issues in the analysis approach or the way that it is being reported, and so that should be made clearer/addressed:

1. Lines 126 to 133: There seems to be confusing descriptions in this section on outcomes. In the first sentence, the authors correctly identify that the primary outcome of the interest in all the key analysis is responsiveness (i.e. the classification as Responder or Non-responder). However, the second sentence reads like it is presenting the secondary aims/objectives of this study, specifically with stating the “association of patient characteristics with responsiveness”. The authors should review to ensure they distinguish outcomes vs objectives.

2. Lines 142 to 143: It is unclear what the authors meant when they say that the same model was used – is it that a) length of stay and 30-day rehospitalizations were added on top of the other variables in the main model in the first part, or b) did only these terms exist in the model, with the patient characteristics and/or main diagnosis/comorbidities removed? Additionally, the authors should justify why two separate models were used in the analysis approach, and whether collinearity checks were performed? Based on the results presented (see point 4 below), this model is not the same if the outcomes of interest are length of stay and 30-day rehospitalizations.

3. Lines 190 to 196: It is unclear in the statistical analysis section of the Methods section how this analysis was performed. This is different than the stratified analysis indicated in the sentence in lines 142 and 143 and so should be explicitly stated. Based on what is reported in Figure 2, there was an interaction term added. This needs to be clearly described and differentiated than if the patient pool was split into two subgroups of those discharged to home or non-home and the same model ran for the two subgroups.

4. Lines 205 to 219: Apart from the lack of clarity in the description of how this analysis was conducted as pointed above in point 2, the phrasing of this section of results in current state indicates that the approach is potentially problematic. Table 2 indicates that the analysis is focused on the outcome of length of stay, discharge to non-home institution, and 30-day rehospitalizations. Additionally, the text reporting the results include phrases like this:

"For responding patients discharged home, length of stay decreased by -0.27 days (95% CI, -0.45 to -0.10), and the likelihood of 30-day rehospitalization was lower by 50% (OR 0.50, 95% CI, 0.45 to 0.56)."

However, the analysis should not be about whether being a responder influences length of stay or not. The length of stay has already occurred for these patients who were surveyed 30 days after discharge. Similarly, if they have any 30-day rehospitalization(s), that event has occurred prior to responding to the phone survey, which was done after 30 days. Responding to the survey is the outcome of interest in the future of these prior healthcare utilization aspects, which are then the factors (not outcomes) that influence the odds of responsiveness.

For all these analyses even for what is included in the supplementary materials, I would suggest that the models be included (they can be indicated together with the tables and figures for those in supplementary materials).

Reviewer 2

Name Simpson, Pippa

Affiliation Medical College of Wisconsin, Pediatrics, Section of Quantitative Health Sciences

Date 14-Aug-2025

COI **None**

Overall, the standard of this paper is higher than average. The findings are important since increasingly whether is the best patient care or not, the medical system wants easy and reliable ways of tracking how resources are to be used and also how patient care can be optimized. However, there is the potential to improve this paper considerably so more focused care can be achieved.

The main issue with this paper is the fact that those who have repeat visits are different from those who do not. The authors have partially considered this in doing a sensitivity analysis by looking at first visit only but have not pursued this further. Clinically this may be an important part since ideally it would be useful the know who might not respond , be discharged incorrectly early or who are likely to return. Statistically, this relates to assumptions about missing data , which in this case are those who do not have repeat hospitalizations and how it relates to the outcomes of interest. Further these morbidity differences are related but in the modelling the interactions or mediating factors are not considered. This is important since in planning it is definitely informative what combinations of morbidity are likely to lead to non-response and also impact the clinical and hospital course.

To summarize , with a large sample size. many factors can be found to be statistically significant. These factors may or may not be clinically significant. Further with a large data set it is possible to consider many factors simultaneously and potentially tease out which are the most important and impact other variables. This was not done. Nor was the potential for missing data to be non-ignorable properly considered.

Line 24: There is a statement of 30 days exactly after admission. Does this mean 30 days after discharge from hospital?

Line 34: the coefficient is not sig diff from 0> there is no explanation.

Line 140: It depends how GEE was done for it to be robust the variables included are not given. . No assumption about missing data is given. Further the correlation structure used is not discussed.

Line 144: It is good that the authors did some sensitivity analysis, but it is limited. No interaction terms are included.

There is some indication that the repeated measure would be more likely to belong to those who were frailer etc. and therefore the results would be different for those that only had one admission from those who had two. This is somewhat clear from the supplementary. In that case some of the assumptions concerning missing data would be violated for responsiveness etc.

Although we are told that the number of calls were limited to 3, the number of calls made is not given nor how they were made, e.g., when in the day / week?

Line 296: What about questions on smart phone. Could look at more aspects, follow up. E.g. Language would not have to be a problem.

Line 321 . It would be easier to consider factors if the interactions were considered.

Page 19, what were the numbers in subgroups?

Page 22: It would be good to see % for those who had one vs more hospitalizations.

Page 29: Similarly, it would be nice to see %

It would be good to see references relating to the issues of missing data.

VERSION 1 - AUTHOR RESPONSE

Reviewer: 1

Dr. Ian Ang, National University Singapore Saw Swee Hock School of Public Health
Comments to the Author:
This study is a secondary analysis of data collected as part of the In-HospiTOOL trial, to assess the factors that are associated with completion of PREMs and PROMs through a phone survey. PREMs and PROMs are important for monitoring and improvement of patient management and care, and so understanding the factors that influence the data collection process is important. The authors provided a strong rationale for this study for which their approach for the most part contain the relevant parts to fulfil the study objectives/research aims. However, there are some potential major issues in the analysis approach or the way that it is being reported, and so that should be made clearer/addressed:
Thank you for this positive assessment. We are happy to address your points.

1. Lines 126 to 133: There seems to be confusing descriptions in this section on outcomes. In the first sentence, the authors correctly identify that the primary outcome of the interest in all the key analysis is responsiveness (i.e. the classification as Responder or Non-responder). However, the second sentence reads like it is presenting the secondary aims/objectives of this study, specifically with stating the "association of patient characteristics with responsiveness". The authors should review to ensure they distinguish outcomes vs objectives.
Thank you for the opportunity to clarify. We have adapted the section accordingly.
Line 137 to 144 "The primary outcome was responsiveness to the survey defined by the question in the survey form stating the interview took place. Secondary outcomes included the association of patient characteristics with reachability by phone and willingness to provide information. Reachability and willingness to provide information was defined by the follow-up question asking for a reason ("patient died", "patient refuses to participate", "patient not reached" or "other") when indicated the interview did not take place. Furthermore, we assessed the association of hospital length of stay, facility discharge and 30-day rehospitalization with responsiveness."

2. Lines 142 to 143: It is unclear what the authors meant when they say that the same

model was used – is it that a) length of stay and 30-day rehospitalizations were added on top of the other variables in the main model in the first part, or b) did only these terms exist in the model, with the patient characteristics and/or main diagnosis/comorbidities removed? Additionally, the authors should justify why two separate models were used in the analysis approach, and whether collinearity checks were performed? Based on the results presented (see point 4 below), this model is not the same if the outcomes of interest are length of stay and 30-day rehospitalizations.

Thank you for this comment. We have clarified the model as follows: Line 153 to 160: "Specifically, we fitted population-averaged logistic regression models with a binomial family, logit link, independent correlation structure, and robust standard errors. To evaluate potential differences in determinants between patients discharged home and those discharged to non-home institutions, we conducted stratified analyses by discharge setting and applied the Wald test of heterogeneity to assess the p-value for interaction. We also conducted analyses to test for associations between facility discharge, length of stay, rehospitalization, and survey responsiveness using the same generalized estimating equations approach."

3. Lines 190 to 196: It is unclear in the statistical analysis section of the Methods section how this analysis was performed. This is different than the stratified analysis indicated in the sentence in lines 142 and 143 and so should be explicitly stated. Based on what is reported in Figure 2, there was an interaction term added. This needs to be clearly described and differentiated than if the patient pool was split into two subgroups of those discharged to home or non-home and the same model ran for the two subgroups.

Thank you for this question and chance to clarify. We used separate subgroup models by discharge location rather than interaction terms, as this approach prioritizes clarity and provides results in a format more easily interpretable for clinicians less familiar with complex statistical modelling. To specify this is the methods section we have added the following sentence (Line 155 to 158): "To evaluate potential differences in determinants between patients discharged home and those discharged to non-home institutions, we conducted stratified analyses by discharge setting and applied the Wald test of heterogeneity to assess the p-value for interaction."

4. Lines 205 to 219: Apart from the lack of clarity in the description of how this analysis was conducted as pointed above in point 2, the phrasing of this section of results in current state indicates that the approach is potentially problematic. Table 2 indicates that the analysis is focused on the outcome of length of stay, discharge to non-home institution, and 30-day rehospitalizations. Additionally, the text reporting the results include phrases like this:

"For responding patients discharged home, length of stay decreased by -0.27 days (95% CI, -0.45 to -0.10), and the likelihood of 30-day rehospitalization was lower by 50% (OR 0.50, 95% CI, 0.45 to 0.56)."

However, the analysis should not be about whether being a responder influences length of stay or not. The length of stay has already occurred for these patients who were surveyed 30 days after discharge. Similarly, if they have any 30-day rehospitalization(s), that event has occurred prior to responding to the phone survey, which was done after 30 days. Responding to the survey is the outcome of interest in the future of these prior healthcare utilization aspects, which are then the factors (not outcomes) that influence the odds of responsiveness.

Thank you for the comment and the opportunity to correct this issue. We agree with your suggestion and have adapted the analysis accordingly. The methods section now reads (Line

158 to 160): "We also conducted analyses to test for associations between facility discharge, length of stay, rehospitalization, and survey responsiveness using the same generalized estimating equations approach."
The results section now includes the following (Line 225 to 230): "Factors associated with responsiveness. Overall, a longer length of stay, facility discharge and rehospitalization within 30 days is associated with decrease in responsiveness. This association is weaker in patients discharged to a non-home setting. (Table 2)."

For all these analyses even for what is included in the supplementary materials, I would suggest that the models be included (they can be indicated together with the tables and figures for those in supplementary materials).
Thank you for this suggestion, we have added the following footnote under the respective figures: "Model specification: Population-averaged logistic regression (xtgee, binomial family, logit link, independent correlation, robust SE)."

Reviewer: 2

Dr. Pippa Simpson, Medical College of Wisconsin
Comments to the Author:
Overall, the standard of this paper is higher than average. The findings are important since increasingly whether is the best patient care or not, the medical system wants easy and reliable ways of tracking how resources are to be used and also how patient care can be optimized. However, there is the potential to improve this paper considerably so more focused care can be achieved.
The main issue with this paper is the fact that those who have repeat visits are different from those who do not. The authors have partially considered this in doing a sensitivity analysis by looking at first visit only but have not pursued this further. Clinically this may be an important part since ideally it would be useful the know who might not respond , be discharged incorrectly early or who are likely to return. Statistically, this relates to assumptions about missing data , which in this case are those who do not have repeat hospitalizations and how it relates to the outcomes of interest. Further these morbidity differences are related but in the modelling the interactions or mediating factors are not considered. This is important since in planning it is definitely informative what combinations of morbidity are likely to lead to non-response and also impact the clinical and hospital course.
To summarize , with a large sample size. many factors can be found to be statistically significant. These factors may or may not be clinically significant. Further with a large data set it is possible to consider many factors simultaneously and potentially tease out which are the most important and impact other variables. This was not done. Nor was the potential for missing data to be non-ignorable properly considered.
Thank you for this valuable input. We fully agree that understanding which patients are likely to return or may be discharged prematurely is clinically very important, especially for patient safety and satisfaction. This aspect has been analyzed extensively in the main In-HospiTOOL study, which examined hospital stay, readmission rate, quality of care, and other clinical outcomes. In the present paper, we specifically focused on survey responsiveness and therefore prioritized clinically relevant variables that would provide interpretable and actionable results for healthcare practice, rather than modeling all possible interactions or mediating factors. To ensure that repeat hospitalizations did not bias our findings, we conducted a sensitivity analysis restricted to patients' first hospitalizations. This analysis

revealed minimal differences, indicating that our results were robust and not driven by repeated measures.

Line 24: There is a statement of 30 days exactly after admission. Does this mean 30 days after discharge from hospital?
Thank you for this comment. The survey was conducted 30 days after hospital admission. We acknowledge that this timing introduces a limitation, as it results in varying lengths of follow-up depending on each patient's length of stay. This could affect the comparability of responses, and we have noted this in the limitations section. Line 338 to 341: "Additionally, surveys were conducted 30 days after hospital admission rather than after discharge, which may affect the comparability of responses due to varying follow-up times depending on patients' length of stay; however, this applied equally to both groups."

Line 34: the coefficient is not sig diff from 0> there is no explanation.
Thank you for this comment, we have adapted the sentence accordingly: Line 32: "A longer length of stay (odds ratio [OR] 0.97; 95% confidence interval [CI], 0.97 to 0.98), discharge to a non-home institution (OR 0.51; 95% CI, 0.47 to 0.54) and rehospitalization within 30 days (OR 0.63; 95% CI, 0.57 to 0.69) is associated with a decreased responsiveness."

Line 140: It depends how GEE was done for it to be robust the variables included are not given. . No assumption about missing data is given. Further the correlation structure used is not discussed.
Thank you for this comment. We have clarified the model as follows (Line 153 to 155): "Specifically, we fitted population-averaged logistic regression models with a binomial family, logit link, independent correlation structure, and robust standard errors."

Line 144: It is good that the authors did some sensitivity analysis, but it is limited. No interaction terms are included.
There is some indication that the repeated measure would be more likely to belong to those who were frailer etc. and therefore the results would be different for those that only had one admission from those who had two. This is somewhat clear from the supplementary. In that case some of the assumptions concerning missing data would be violated for responsiveness etc.
Thank you for this question and the opportunity to clarify. We appreciate your concern regarding missing data. In our study, all contacted patients were categorized as having completed the survey, explicitly refused, could not be reached after repeated attempts, or were deceased, ensuring that no traditional missing data issue arises. Furthermore, we analyzed each hospitalization separately and did not compare first versus subsequent hospitalizations. Had we intended to compare different hospitalizations of the same patient, a missing data issue would indeed have emerged, as only a minority of patients experienced more than one hospitalization. This approach allowed us to present robust and complete results for each hospitalization.

Although we are told that the number of calls were limited to 3, the number of calls made is not given nor how they were made, e.g., when in the day / week?
Thank you for this question. We believe patient responsiveness was not influenced by more than three call attempts, which were made at different times of the day and on different days to maximize reachability. If a patient refused participation, this decision was always respected, and no further attempts were made. In cases where patients could not be reached after three attempts, the outcome was documented as "not reached." This standardized

approach ensured consistency across interviewers and minimized the risk of undue pressure on patients.

Line 296: What about questions on smart phone. Could look at more aspects, follow up. E.g. Language would not have to be a problem.
Thank you very much for this comment. We agree that smartphone apps or similar methods could help overcome language barriers; however, we deliberately limited data collection to a single method to ensure comparability. Given our multimorbid and mostly older population, telephone interviews were considered the most appropriate approach, as they also allowed clarification of questions when needed. We have added smartphones as an option in the discussion section (Line 330)

Line 321 . It would be easier to consider factors if the interactions were considered.
Thank you for this important comment. We agree that considering interactions can in principle provide additional insights; however, we deliberately chose not to pursue this in the current analysis for several reasons. First, including multiple interaction terms would greatly increase model complexity and make interpretation more difficult, especially since our primary aim was to estimate overall associations rather than to explore every possible subgroup. Second, adding numerous interactions inflates the risk of type I error due to multiple testing, particularly when such interactions are not pre-specified. Third, interaction models require larger effective sample sizes in each stratum to obtain stable estimates, which can be challenging even in large datasets if some subgroups are small. Finally, we sought to maintain clarity and ensure that our findings remain understandable and applicable for a broad clinical audience; focusing heavily on complex interaction models would have shifted the paper's focus away from its primary research question and made the results more difficult to communicate to practicing physicians.

Page 19, what were the numbers in subgroups?
Thank you very much for this question. We initially decided not to include tables for all subgroups in order to avoid overloading the manuscript with data. However, we are happy to add them as supplementary material. Additionally we have added the following sentence in the results section: Line 211: "Baseline characteristics for patients discharged to home and patients to non-home settings can be found in Tables S2-3."

Page 22: It would be good to see % for those who had one vs more hospitalizations.
Thank you for this comment. We have added this information to the baseline.

Page 29: Similarly, it would be nice to see %
Thank you for this remark, we have added this information to the baseline.

It would be good to see references relating to the issues of missing data.
Thank you for the opportunity to clarify on missing data, we have done so for the question above.

VERSION 2 - REVIEW

**Reviewer**	**1**
**Name**	**Ang, Ian**

Affiliation **National University Singapore Saw Swee Hock School of Public Health**

Date **17-Sep-2025**

COI

The authors did a great job in improving the clarity of their manuscript and in addressing the various comments/questions from both peer reviewers. There are still some points to address to further improve the manuscript:

1. Lines 120 to 127: This section is still confusing. The issue lies in the choice of word "outcome" which instinctively refers to the outcome VARIABLES of interest. This would be different if the authors are choosing to use this section to list Research Objectives/Aims. If it is the first, then I would still suggest removing "the association of patient characteristics with" in the sentence in Lines 122 to 123. The secondary outcome variables of interest are thus phone reachability and willingness to provide information, while the study objective/aim is to assess the factors associated with these outcomes. Lines 126 to 127 are redundant then. Alternatively, if the authors want to keep to describing study objectives to keep the existing bulk of content, then the section header and the term should be changed to study objectives/aims. And the first sentence (lines 121 to 122) should be rephrased to be about the main analysis instead of just a description of the variable.

2. Lines 162 to 171 and Table 1: The analysis should present the length of stay, especially since that is included in the subsequent analysis of factors associated with likelihood of survey response. Now that it has been clarified that patients were contacted to complete the survey 30 days counting from admission date (and not discharge date), it would also be important to provide the descriptive of the proportion of patients in each group of responders vs non-responders that were still in the hospital at this 30-day mark – this could either be that they were still hospitalized from that index admission only, or even if including any subsequent rehospitalization. This might be the most important factor to consider – if a patient is still hospitalized, they would not likely answer phone calls and/or be in physical/mental state suitable for responding to phone surveys.

3. Table 1: "Number of Hospitalizations" – unclear what is the duration of tracking period. Is this only 30 days? Might be clearer to indicate the category as number of 30-day readmissions with 1st category as 0?

4. Lines 206 to 208 (and 141 to 143): Great that the analysis has been updated. First to clarify, the "Rehospitalization within 30 days" is 30 days after discharge, right? More importantly, was it coded as a binary Yes-No of any rehospitalization within 30 days? Or was it treated as a continuous count data. Line 34 in Abstract does not indicate rates, while Line 235 in Discussion does. Would be good to align the language to more instinctively reflect the analysis.

5. Table 2: Use consistent spelling of hospitalization with “z”.

Reviewer **2**

Name **Simpson, Pippa**

Affiliation **Medical College of Wisconsin, Pediatrics, Section of Quantitative Health Sciences**

Date **09-Sep-2025**

COI

The authors have clearly spent some time and thought on their responses. However, the responses show some incorrect reasoning (and analysis). Several of the erroneous responses they gave are perhaps due to a lack of a (PhD) biostatistician on their team. The reviewer would recommend their consultation with such a person. Despite this with the correct analysis the conclusions of this paper would probably remain very similar.

Below the reviewer has taken "the response the authors" made and has responded showing where the faulty statistical reasoning may lie .

"we fitted population-averaged logistic regression models with a binomial family, logit link, independent correlation structure, and robust standard errors.”

As stated, this assumes that the repeated measures are independent. Since it is clear that this is not the case because repeat hospitalizations are different in their behavior, the model would be incorrect.

"Thank you for this question and the opportunity to clarify. We appreciate your concern regarding missing data. In our study, all contacted patients were categorized as having completed the survey, explicitly refused, could not be reached after repeated attempts, or were deceased, ensuring that no traditional missing data issue arises. Furthermore, we analyzed each hospitalization separately and did not compare first versus subsequent hospitalizations. Had we intended to compare different hospitalizations of the same patient, a missing data issue would indeed have emerged, as only a minority of patients experienced more than one hospitalization. This approach allowed us to present robust and complete results for each hospitalization."

The authors do not seem to understand that they do indeed have missing data, since some people have two or more measurements and others only have one. If they had looked at some references relevant to this topic they should have realized this mistake. As before, a reference to why an exploration of the implications of missing data should be included as a reference or otherwise and mentioned in the methods. In making the decision to allow some patients to have more than one observation (response for a different hospitalization) they should allow for the type of missing data. If the authors are going to assume that the data are missing at random (a typical assumption which is usually manageable and reasonable)

the authors would have to do maximum likelihood estimation to take account of this assumption. If they did, they should state this in the description of the model. In any case, they should include a list of variables included in their respective models.

A check of the assumptions about missingness was in effect done as a sensitivity analysis. In the authors' sensitivity analysis, the first observation (hospitalization) only was used and there was indeed a somewhat different result suggesting that response could be associated at least partly with whether a patient was rehospitalized or not. The reviewer calculated that there are 5305 who are rehospitalized and 14947 who have only one visit. This means that if only main effects are looked at the first visit will dominate results (as it does). Thus, although there are differences in the analysis of first visit and of all mainly agree, this is not too surprising. Further analysis needs to be done if the response rate of those who have to be rehospitalized is to be investigated.

Another good exploration of the missing data is to use missing (only first hospitalization) vs not more than one) as an outcome and see if in fact some of the variables you included are significant for missingness.

"Thank you for this important comment. We agree that considering interactions can in principle provide additional insights; however, we deliberately chose not to pursue this in the current analysis for several reasons. First, including multiple interaction terms would greatly increase model complexity and make interpretation more difficult, especially since our primary aim was to estimate overall associations rather than to explore every possible subgroup. Second, adding numerous interactions inflates the risk of type I error due to multiple testing, particularly when such interactions are not pre-specified."

First, philosophically exploring associations does include looking at how responses vary subject to possible mediating or confounding variables, which may be interactions. The sample size is certainly large enough. Subset analysis has the disadvantage of not really providing a comparison for one subset against the other but in the case being discussed it may be better than including interactions in a generalized linear model because for example, the response pattern for those with a longer hospital stay, may also be associated with a readmission, whereas the response for those not staying longer might be associated with being admitted to a facility other than home. For this paper to have impact it is important to know where to target interventions to get the information required.

Classification trees would be an effective way to look at how response depends on the interrelationships of the outcome and other variables. and provide a transparent model. To use these, I would restrict the analysis, at first, to the first visit only. But repeated measures can be accommodated. Since the appropriate method for trees would be a validation approach – and the sample is more than adequate to do this – this might be a stronger and better model than the one used and the point about type 1 error not relevant.

VERSION 2 - AUTHOR RESPONSE

<u>Reviewer: 2</u>
Dr. Pippa Simpson, Medical College of Wisconsin
Comments to the Author:
The authors have clearly spent some time and thought on their responses. However, the responses show some incorrect reasoning (and analysis). Several of the erroneous responses they gave are perhaps due to a lack of a (PhD) biostatistician on their team. The reviewer would recommend their consultation with such a person. Despite this with the correct analysis the conclusions of this paper would probably remain very similar. Below the reviewer has taken "the response the authors" made and has responded showing where the faulty statistical reasoning may lie .
"we fitted population-averaged logistic regression models with a binomial family, logit link, independent correlation structure, and robust standard errors."
As stated, this assumes that the repeated measures are independent. Since it is clear that this is not the case because repeat hospitalizations are different in their behavior, the model would be incorrect.
Thank you for this valuable comment and for identifying the issue with our original model specification. You are correct that our initial approach, which used an independent correlation structure, did not appropriately account for within-patient correlation among repeated hospitalizations.

We have now corrected this. We re-fitted the primary outcome models using generalized estimating equations (GEE) with a binomial family, logit link, and an exchangeable working correlation structure. This specification correctly accounts for the non-independence of repeated observations from the same patient. Robust (sandwich) standard errors were used to ensure valid inference even if the working correlation is not perfectly specified.
We selected the exchangeable structure, which is appropriate when the number and spacing of repeated hospitalizations vary between patients. An autoregressive (AR1) structure would require approximately equal time intervals between measurements, which is not the case in our data, as hospitalization timing is highly irregular.

Initially, we employed the Stata program developed by Cui and Qian to identify the most appropriate working correlation structure for our GEE analyses (Cui & Qian, 2007). Based on the results of this procedure, we initially selected an independent correlation structure. However, more recent literature has noted that: "Note that QIC(R) and QIC(R) HH do not perform well in distinguishing the independence and exchangeable "working" correlation structures because, in certain cases, the same regression parameter estimates can be obtained under these two structures."(Wang, 2014). The exchangeable correlation therefore provides a reasonable way to model within-patient correlation while maintaining model stability and interpretability.

We attempted to fit the secondary outcome model for willingness to provide information with an exchangeable correlation structure as well; however, estimation failed with "correlation > 1," reflecting instability due to the large proportion of patients with only a single hospitalization. We therefore used an independent working correlation structure with robust (sandwich) standard errors, which provide valid population-averaged inference even when the true correlation is nonzero.

We have adapted the methods section accordingly (LINE 134-138): Specifically, we fitted population-averaged logistic regression models with a binomial family, logit link, exchangeable correlation structure, and robust standard errors. For the willingness to

provide information outcome, we used an independent correlation structure due to estimation failure caused by a large proportion of patients with only one hospitalization."

"Thank you for this question and the opportunity to clarify. We appreciate your concern regarding missing data. In our study, all contacted patients were categorized as having completed the survey, explicitly refused, could not be reached after repeated attempts, or were deceased, ensuring that no traditional missing data issue arises. Furthermore, we analyzed each hospitalization separately and did not compare first versus subsequent hospitalizations. Had we intended to compare different hospitalizations of the same patient, a missing data issue would indeed have emerged, as only a minority of patients experienced more than one hospitalization. This approach allowed us to present robust and complete results for each hospitalization."

The authors do not seem to understand that they do indeed have missing data, since some people have two or more measurements and others only have one. If they had looked at some references relevant to this topic they should have realized this mistake. As before, a reference to why an exploration of the implications of missing data should be included as a reference or otherwise and mentioned in the methods. In making the decision to allow some patients to have more than one observation (response for a different hospitalization) they should allow for the type of missing data. If the authors are going to assume that the data are missing at random (a typical assumption which is usually manageable and reasonable) the authors would have to do maximum likelihood estimation to take account of this assumption. If they did, they should state this in the description of the model. In any case, they should include a list of variables included in their respective models.

Thank you for this careful and helpful comment explaining our missunderstanding.
As an additional sensitivity analysis, we fitted a random-intercept logistic regression model (melogit), which provides subject-specific (conditional) estimates using maximum-likelihood estimation. The results were directionally consistent with the GEE estimates, although—as expected—subject-specific effects showed larger confidence intervals.

We have decided to keep the GEE model as main analysis and add the melogit as a second sensitivity analysis, as our primary research objective was to estimate average effects at the hospitalization level across the patient population, rather than to model within-patient changes. The GEE framework provides these population-averaged estimates, which are more directly relevant for informing system-level and policy interventions. In contrast, mixed-effects models such as melogit yield subject-specific estimates that are more appropriate when the research question concerns individual-level trajectories.

We have added the follwing part in the methods section (LINE 143-148): "Additionally, we performed two sensitivity analyses: first, we restricted the sample to each patient's first hospitalization during the study period and fitted population-averaged logistic regression models with a binomial family, logit link, with an independent correlation structure; second, we fitted mixed-effects logistic regression models including all hospitalizations with a random intercept for each patient to account for within-patient correlation, using maximum likelihood estimation."

Forthermore, this was added to the results section (LINE 232-234): "Results from the second sensitivity analysis using mixed-effects logistic regression, accounting for within-patient

correlation, were consistent with the main findings, although confidence intervals were wider, further supporting the robustness of the results. (eFigures 7-10)"

A check of the assumptions about missingness was in effect done as a sensitivity analysis. In the authors' sensitivity analysis, the first observation (hospitalization) only was used and there was indeed a somewhat different result suggesting that response could be associated at least partly with whether a patient was rehospitalized or not. The reviewer calculated that there are 5305 who are rehospitalized and 14947 who have only one visit. This means that if only main effects are looked at the first visit will dominate results (as it does). Thus, although there are differences in the analysis of first visit and of all mainly agree, this is not too surprising. Further analysis needs to be done if the response rate of those who have to be rehospitalized is to be investigated.

Another good exploration of the missing data is to use missing (only first hospitalization) vs not more than one) as an outcome and see if in fact some of the variables you included are significant for missingness.
We appreciate this thoughtful suggestion. While the proposed analysis is statistically reasonable, its clinical interpretation would be ambiguous. Patients with missing data (no rehospitalization) may represent a heterogeneous group — including those who died, relocated, sought care elsewhere, or simply did not require another admission. Because these reasons cannot be distinguished with our available data, and as analyses of factors leading to rehospitalization have been addressed in other studies, we considered this question beyond the scope of our work, which focuses on patient responsiveness to post-discharge surveys.

"Thank you for this important comment. We agree that considering interactions can in principle provide additional insights; however, we deliberately chose not to pursue this in the current analysis for several reasons. First, including multiple interaction terms would greatly increase model complexity and make interpretation more difficult, especially since our primary aim was to estimate overall associations rather than to explore every possible subgroup. Second, adding numerous interactions inflates the risk of type I error due to multiple testing, particularly when such interactions are not pre-specified."

First, philosophically exploring associations does include looking at how responses vary subject to possible mediating or confounding variables, which may be interactions. The sample size is certainly large enough. Subset analysis has the disadvantage of not really providing a comparison for one subset against the other but in the case being discussed it may be better than including interactions in a generalized linear model because for example, the response pattern for those with a longer hospital stay, may also be associated with a readmission, whereas the response for those not staying longer might be associated with being admitted to a facility other than home. For this paper to have impact it is important to know where to target interventions to get the information required.
We thank the reviewer and agree that subset analyses are appropriate; they allow meaningful comparisons across key patient groups, are easier to interpret for our target audience, and align with our primary aim of estimating overall associations rather than exploring every possible subgroup.

Classification trees would be an effective way to look at how response depends on the

interrelationships of the outcome and other variables. and provide a transparent model. To use these, I would restrict the analysis, at first, to the first visit only. But repeated measures can be accommodated. Since the appropriate method for trees would be a validation approach – and the sample is more than adequate to do this – this might be a stronger and better model than the one used and the point about type 1 error not relevant.

We thank the reviewer for this insightful suggestion. We agree that classification trees could provide a transparent and interpretable approach to exploring how survey response depends on multiple variables and their interrelationships. While this is a promising alternative, we focused our current analysis on population-averaged associations using GEE to align with our primary study aim of estimating overall effects rather than building predictive models.

We acknowledge that classification trees, particularly when restricted to the first visit or combined with a validation approach, could be a valuable tool in future work to explore complex patterns and interactions in survey response.

<u>Reviewer: 1</u>

Dr. Ian Ang, National University Singapore Saw Swee Hock School of Public Health

Comments to the Author:

The authors did a great job in improving the clarity of their manuscript and in addressing the various comments/questions from both peer reviewers. There are still some points to address to further improve the manuscript:

Thank you very much.

1. Lines 120 to 127: This section is still confusing. The issue lies in the choice of word "outcome" which instinctively refers to the outcome VARIABLES of interest. This would be different if the authors are choosing to use this section to list Research Objectives/Aims. If it is the first, then I would still suggest removing "the association of patient characteristics with" in the sentence in Lines 122 to 123. The secondary outcome variables of interest are thus phone reachability and willingness to provide information, while the study objective/aim is to assess the factors associated with these outcomes. Lines 126 to 127 are redundant then. Alternatively, if the authors want to keep to describing study objectives to keep the existing bulk of content, then the section header and the term should be changed to study objectives/aims. And the first sentence (lines 121 to 122) should be rephrased to be about the main analysis instead of just a description of the variable.

Thank you for this comment. We have adapted the section accordingly (LINE 121-125): "The primary outcome was responsiveness to the survey defined by the question in the survey form stating the interview took place. Secondary outcomes included reachability by phone, and willingness to provide information, defined by the follow up question asking for a reason ("patient died", "patient refuses to participate", "patient not reached" or "other") when indicated the interview did not take place."

2. Lines 162 to 171 and Table 1: The analysis should present the length of stay, especially since that is included in the subsequent analysis of factors associated with likelihood of survey response. Now that it has been clarified that patients were contacted to complete the survey 30 days counting from admission date (and not discharge date), it would also be important to provide the descriptive of the proportion of patients in each group of responders vs non-responders that were still in the hospital at this 30-day mark – this

could either be that they were still hospitalized from that index admission only, or even if including any subsequent rehospitalization. This might be the most important factor to consider – if a patient is still hospitalized, they would not likely answer phone calls and/or be in physical/mental state suitable for responding to phone surveys.
Thank you for this valuable comment, we have added the length of stay to the baseline (Table 1) and added the sentence to the results section (LINE 171-173): "Responders had a median length of stay of 6 days (IQR 3–9), compared with 6 days (IQR 4–10) for non-responders (SMD 0.14)."

3. Table 1: "Number of Hospitalizations" – unclear what is the duration of tracking period. Is this only 30 days? Might be clearer to indicate the category as number of 30-day readmissions with 1st category as 0?
Thank you for this question. The measure refers to the entire study period. It was included primarily to provide context for the sensitivity analysis, which considers only the first hospitalization for each patient.

4. Lines 206 to 208 (and 141 to 143): Great that the analysis has been updated. First to clarify, the "Rehospitalization within 30 days" is 30 days after discharge, right? More importantly, was it coded as a binary Yes-No of any rehospitalization within 30 days? Or was it treated as a continuous count data. Line 34 in Abstract does not indicate rates, while Line 235 in Discussion does. Would be good to align the language to more instinctively reflect the analysis.
Thank you for this important question. The Swiss Federal Office for Statistics nationwide inpatient claims database included a variable called "days until next hospitalization," which we used to define 30-day rehospitalization as a binary variable.
Additionally, we revised the sentence in the Discussion section to clarify that we used a binary variable and calculated odds ratios (LINE 240-243): "Responders had shorter hospital stays, were less likely to be discharged to a non-home institution and had a lower relative risk for 30-day rehospitalization compared to non-responders, particularly among those discharged home."

5. Table 2: Use consistent spelling of hospitalization with "z".
Thank you for bringing this to our attention, we have corrcted the mistake.

Bibliography

Cui, J., & Qian, G. (2007). Selection of Working Correlation Structure and Best Model in GEE Analyses of Longitudinal Data. *Communications in Statistics - Simulation and Computation, 36*(5), 987-996. doi:10.1080/03610910701539617

Wang, M. (2014). Generalized Estimating Equations in Longitudinal Data Analysis: A Review and Recent Developments. *Advances in Statistics, 2014*(1), 303728. doi:https://doi.org/10.1155/2014/303728

VERSION 3 - REVIEW

Reviewer 1

Name **Ang, Ian**

Affiliation **National University Singapore Saw Swee Hock School of Public Health**

Date **20-Nov-2025**

COI

The authors further improved the clarity of their manuscript and in addressing the various comments/questions from both peer reviewers. I have no further questions or issues to be addressed.

Reviewer **2**

Name **Simpson, Pippa**

Affiliation **Medical College of Wisconsin, Pediatrics, Section of Quantitative Health Sciences**

Date **16-Nov-2025**

COI

Thank you for answering almost all my concerns. A limitation of all your analysis (and it is unavoidable really) is the assumption that the data were missing at random and the sensitivity analysis partially addressed that.
